# Assessment of a Novel VEGF Targeted Agent Using Patient-Derived Tumor Tissue Xenograft Models of Colon Carcinoma with Lymphatic and Hepatic Metastases

**DOI:** 10.1371/journal.pone.0028384

**Published:** 2011-12-02

**Authors:** Ketao Jin, Guangliang Li, Binbin Cui, Jing Zhang, Huanrong Lan, Na Han, Bojian Xie, Feilin Cao, Kuifeng He, Haohao Wang, Zhenzhen Xu, Lisong Teng, Tieming Zhu

**Affiliations:** 1 Department of Surgical Oncology, First Affiliated Hospital, College of Medicine, Zhejiang University, Hangzhou, Zhejiang, China; 2 Department of Surgery, Affiliated Zhuji Hospital, Wenzhou Medical College, Zhuji, Zhejiang, China; 3 Department of Surgical Oncology, Taizhou Hospital, Wenzhou Medical College, Taizhou, Zhejiang, China; 4 Cancer Chemotherapy Center, Zhejiang Provincial Cancer Hospital, Zhejiang University of Traditional and Chinese Medicine, Hangzhou, Zhejiang, China; Huntsman Cancer Institute, University of Utah, United States of America

## Abstract

The lack of appropriate tumor models of primary tumors and corresponding metastases that can reliably predict for response to anticancer agents remains a major deficiency in the clinical practice of cancer therapy. It was the aim of our study to establish patient-derived tumor tissue (PDTT) xenograft models of colon carcinoma with lymphatic and hepatic metastases useful for testing of novel molecularly targeted agents. PDTT of primary colon carcinoma, lymphatic and hepatic metastases were used to create xenograft models. Hematoxylin and eosin staining, immunohistochemical staining, genome-wide gene expression analysis, pyrosequencing, qRT-PCR, and western blotting were used to determine the biological stability of the xenografts during serial transplantation compared with the original tumor tissues. Early passages of the PDTT xenograft models of primary colon carcinoma, lymphatic and hepatic metastases revealed a high degree of similarity with the original clinical tumor samples with regard to histology, immunohistochemistry, genes expression, and mutation status as well as mRNA expression. After we have ascertained that these xenografts models retained similar histopathological features and molecular signatures as the original tumors, drug sensitivities of the xenografts to a novel VEGF targeted agent, FP3 was evaluated. In this study, PDTT xenograft models of colon carcinoma with lymphatic and hepatic metastasis have been successfully established. They provide appropriate models for testing of novel molecularly targeted agents.

## Introduction

Animal models have been used in front-line preclinical studies for predicting efficacy and possible toxicities of anticancer drugs in cancer patients [1]. Advancing a laboratory candidate drug from preclinical testing into testing in phase II clinical trials is based on the assumption that cancer models used in the laboratory are clinically predictive [2]. One of the most serious obstacles confronting investigators involved in the development and assessment of new anticancer drugs is the failure of rodent tumor models to predict reliably as to whether a given drug will have prospective anticancer activity with acceptable toxicity when applied to humans. Current tumor models used for drug evaluation generally consist of implantation into immunodeficient mice of xenografts generated from well-established human cancer cell lines that have already adapted to in vitro growth. These models have been used extensively for decades for rapid screening of the anticancer drug efficacy [3], [4]. Such models have proven useful for identifying cellular and molecular mechanisms underlying metastasis and for developing new therapeutics. However, limited effectiveness exists, which severely restrains the predictive power of such models assessing the responses of patients' tumors to anticancer drugs in the clinic. The highly anaplastic cancer cells cultivated in vitro represent the extreme derivates from highly advanced cancers and are not associated with original tumor stroma, which now has been recognized as a crucial factor in the pathogenesis of cancer metastasis. In recent years, various groups have initiated the development of more relevant models based on xenografting of primary human tumor tissue in immunodeficient mice. Such patient-derived tumor tissue (PDTT) xenograft models are mainly constructed by introducing advanced tumor cells into the subcutaneous graft site. These xenografts models retain similar morphology, architecture and molecular signatures as the original cancers and thus should be used for rapid screening of potential therapeutics.

In recent years, many studies have focused on the heterogeneity found in primary tumors and corresponding metastases with the consideration that evaluation of metastatic rather than primary sites could be of clinical relevance [5]. Numerous reports have evaluated the heterogeneity in primary tumors and corresponding metastases in a range of solid tumors such as breast cancer [6], [7], [8], [9], [10], [11], [12], [13], colorectal cancer [14], [15], [16], [17] and non-small cell lung cancer (NSCLC) [18], [19]. The main purpose of investigating the heterogeneity found in primary tumors and corresponding metastases is to evaluate the effect of such heterogeneity on the efficacy of anticancer therapy and cancer patients' prognosis. The primary tumor and its corresponding metastases are different at the molecular marker expression or gene status levels and that these differences may affect the clinical outcome of anticancer therapy [20]. Monaco et al. suggested that the *EGFR* and *KRAS* status of primary lung carcinomas might not predict the status in the corresponding metastases. Their observation may have important implications for molecular testing for EGFR-targeted therapies [21]. A retrospective study investigated the role of PTEN loss, Akt phosphorylation and *KRAS* mutations in primary colorectal tumors and their corresponding metastases on the activity of cetuximab plus irinotecan [22]. This study gave us direct evidence to reveal that the genetic heterogeneity in primary colorectal tumors and their corresponding metastases have different responses to EGFR-targeted therapy. On these considerations mentioned above, to establish the PDTT xenograft models of primary tumor and corresponding metastases for assessment of the response of both the primary tumor and the metastases to some novel drugs is extremely useful.

FP3 (also named as KH902 or KH903) is an engineered protein which contains the extracellular domain 2 of VEGF receptor 1 (Flt-1) and extracellular domain 3 and 4 of VEGF receptor 2 (Flk-1, KDR) fused to the Fc portion of human immunoglobulin G1 [23], [24]. Previous studies indicated that FP3 had promise as a local antiangiogenic treatment of human CNV (choroidal neovascularization) -related AMD (age-related macular degeneration) [23], [25], [26], [27]. In subsequent studies, it was demonstrated that FP3 has an inhibitory efficacy in VEGF-mediated proliferation and migration of human umbilical vein endothelial cells, and in VEGF-mediated vessel sprouting of rat aortic ring *in vitro* [24]. It was also demonstrated that FP3 has an antitumor effect in a NSCLC cell line (A549) xenograft model [24] and a PDTT xenograft model of gastric carcinoma [28] in nude mice.

It was the aim of our study to establish PDTT xenograft models of colon carcinoma with lymphatic and hepatic metastasis useful for testing of a novel molecularly targeted agent, FP3.

## Materials and Methods

### Reagents and drugs

Anti-Akt, anti-ERK, anti-MAPK and anti-mTOR antibodies, and phosphorylation-specific antibodies against Akt (Ser^308^ and Ser^473^), ERK (Thr^202^/Tyr^204^), MAPK (Thr^180^/Tyr^182^) and mTOR (Ser^2448^) were purchased from Cell Signaling Technology Inc. (Cell Signaling, Beverly, MA). The antibodies against cleaved-caspase-3, VEGFR-2, and PCNA were purchased from Cell Signaling Technology Inc. (Cell Signaling, Beverly, MA). The antibodies against CK-20 (human-specific) and CDX-2 (human-specific) were purchased from DAKO (Carpinteria, CA). The antibodies against VEGF (human-specific) and EGFR were purchased from Epitomics Inc. (Burlingame, CA). The antibody against platelet endothelial cell adhesion molecule-1 (PECAM-1, CD31) (rat monoclonal, clone MEC 13.3) was purchased from BD Pharmingen (San Diego, CA). The antibody against α-smooth muscle actin (α-SMA, rabbit polyclonal) was purchased from Abcam (Cambridge, UK). Fluorescent (Cy3- or FITC-conjuncted) secondary antibodies (goat anti-rat or anti-rabbit) were purchased from Jackson ImmunoResearch (West Grove, PA). The antibody against GAPDH was purchased from Santa Cruz Biotechnology, Inc. (Santa Cruz, CA). Horseradish peroxidase-conjugated secondary antibodies were purchased from Santa Cruz Biotechnology, Inc. (Santa Cruz, CA). Chemiluminescent detection system was purchased from Amersham Pharmacia Biotech (Arlington Heights, IL). RPMI 1640 medium, fetal bovine serum (FBS), penicillin, and streptomycin were purchased from Gibco (Grand Island, NY, USA). Isofluorane, diethyl ether, ketamine, and xylazine were purchased from Sigma (St. Louis, MO, USA). Bevacizumab (Avastin) was purchased from Roche, Inc. (Roche, USA). FP3 was kindly provided as a gift from Kanghong, Biotechnology Inc. (Konghong, Chengdu, China).

### Patient and tissue samples

Tumor specimens were obtained at initial surgery from a 40-year-old female colon carcinoma patient with lymphatic and hepatic metastases. Prior written informed consent was obtained from the patient and the study received ethics board approval at First Affiliated Hospital, College of Medicine, Zhejiang University. The patient had not received chemotherapy or radiation therapy before surgery. The histological type was determined according to WHO criteria. The tumor was diagnosed as mucinous adenocarcinoma (T3N2M1). The tumor samples of colon carcinoma with lymphatic and hepatic metastases were put into medium immediately after surgical resection under sterile conditions and transported without delay to the animal facility.

### Establishment of xenografts

Four- to six-week-old female BALB/c nude mice purchased from Slaccas (Slaccas Laboratory Animal, Shanghai, China) were housed in a barrier facility and acclimated to 12-h light-dark cycles for at least three days before use. The use of experimental animals adhered to the “Principles of Laboratory Animal Care” (NIH publication #85-23, revised in 1985). All experiments were approved by the Institutional Animal Care and Use Committee of Zhejiang University (approval ID: SYXK(ZHE)2005-0072). The method to establish the PDTT xenograft models of human colon carcinoma with lymphatic and hepatic metastases were followed as described previously [5], [28], [29], [30]. Briefly, tumors were placed in RPMI 1640 medium supplemented with 20% FBS and 0.05% penicillin/streptomycin solution in an ice bath in the surgical site. Tumors were then transferred to a sterile Petri dish containing RPMI 1640 medium with supplements mentioned above. Thin slices of tumor were diced into 2×2×3 mm^3^ pieces and washed thrice with RPMI 1640 with supplements mentioned above. Under anesthesia with isofluorane, tumors were implanted into 4- to 6-week-old female athymic nude mice by a small incision and subcutaneous pocket made in one side of the lower back in which one tumor piece is deposited in the pocket. While the pocket was still open, one drop of 100× penicillin/streptomycin solution was placed into the opening. Half of the rest of the tumors was cryopreserved in liquid nitrogen and the other half was immediately snap-frozen and stored at −80°C and processed for biological studies, such as genetic, genomic, mRNA expression and protein expression analyses. For each tumor, twenty mice were used. Growth of established tumor xenografts was monitored at least twice weekly by vernier caliper measurement of the length (a) and width (b) of tumor. Tumor volumes was calculated as (a×b^2^)/2. For the first week following implantation, a small bump would be visible where the tumor was inserted. At 14 to 22 weeks following implantation, a tumor began to appear at the site of implantation with 1000 to 1500 mm^3^ in volume. At a size of about 1500 mm^3^, tumors were removed for serial transplantation. Tumor-bearing animals were anesthetized with diethyl ether and sacrificed by cervical dislocation. Animals were placed immediately in an ice water bath for 2 minutes. The mice were then placed in 75% ethanol for 2 minutes, and transferred to a laminar flow hood for dissection. Tumors were minced under sterile conditions and implanted in successive nude mice as described above. Tumors were passaged no more than 10 times. Following transplantation, tumors were allowed to grow to 200 to 500 mm^3^ before initiation of treatment for drug evaluation. Numerous samples from early passages were stored in the tissue bank and cryopreserved in liquid nitrogen, and used for further experiments.

### Treatment protocol

Xenografts from this second mouse-to-mouse passage (the third generation, G3) were allowed to grow to a size of 200 mm^3^, at which time mice were randomized in the following three groups of treatment, with 10 mice in each group: (a) control (100 µl saline); (b) FP3, 15 mg/kg, i.v., twice per week; (c) Avastin (bevacizumab), 10 mg/kg, i.v., twice per week. Mice were treated during 21 days, monitored twice per week for signs of toxicity, and were weighed once a week. Tumor size was evaluated twice a week by caliper measurements using the following formula: tumor volume = (length×width^2^)/2. Relative tumor growth inhibition (TGI) was calculated by relative tumor growth of treated mice divided by relative tumor growth of control mice (T/C). Experiments were terminated on day 30.

### Histology and immunohistochemistry

Selected tumor specimen were fixed in 10% neutral-buffered formalin and embedded in paraffin. Five-micrometer sections were cut, dewaxed, and then rehydrated and stained with hematoxylin and eosin (H&E) as described previously [31], [32].

For immunohistochemical staining, five micromolar sections were cut, dewaxed, rehydrated, and subjected to antigen retrieval. After blocking endogenous peroxidase activity, the sections were incubated with the primary antibodies against CK-20 (1∶100), CDX-2 (1∶100), EGFR (1∶100), VEGF (1∶100) and PCNA (1∶100) (overnight at 4°C). Immunohistochemistry was performed using the streptavidin-biotin peroxidase complex method (Lab Vision, Fremont, CA). The slides were examined and pictures were taken using an Olympus BX60 (Olympus, Japan). Sections known to stain positively were incubated in each batch and negative controls were also prepared by replacing the primary antibody with preimmune sera.

### Fluorescent immunohistochemistry

Selected mice with similar tumor size were anesthetized with ketamine (87 mg/kg) plus xylazine (13 mg/kg) injected intramuscularly. The chest was opened rapidly, and the vasculature was perfused for 3 minutes at a pressure of 120 mmHg with fixative [4% paraformaldehyde in 0.1 mol/L phosphate-buffered saline (PBS), pH 7.4] from an 18-gauge cannula inserted into the aorta via an incision in the left ventricle. Blood and fixative exited through an opening in the right atrium. After the perfusion, the implanted tumor was removed and placed into fixative for 2 hours at 4°C. Specimens were then rinsed several times with PBS, infiltrated overnight with 30% sucrose, embedded in OCT medium and frozen for cryostat sectioning [33]. Cryostat sections 8 to 10 µm in thickness were brought to room temperature, air dried overnight, then fixed in acetone for 10 min. Slides were allowed to air dry for 30 min and were washed three times for 5 min each in PBS. Samples were then incubated in 5% BSA in PBS for 30 min at room temperature to block nonspecific antibody binding. Next, the sections were incubated with two primary antibodies (CD31, 1∶100; and α-SMA, 1∶200) or VEGFR-2 (1∶150) overnight at room temperature in humidified chambers diluted in PBS. After several rinses with PBS, specimens were incubated for 1 hour at room temperature with fluorescent (Cy3- or FITC-conjuncted) secondary antibodies (goat anti-rat or goat anti-rabbit) diluted (1∶200) in PBS. Specimens were rinsed again with PBS, and mounted in Vectashield (Vector Laboratories, Burlingame, CA) [34], [35]. Tissue sections were examined and digitally photographed using a Zeiss Axiophot fluorescence microscope (Carl Zeiss, Thornwood, NY) equipped with single, dual, and triple fluorescence filters and a low-light, externally cooled, three-chip charge-coupled device (CCD) camera (480×640 pixel RGB-color images, CoolCam; SciMeasure Analytical Systems, Atlanta, GA) and saved as TIFF files.

### Genome-wide gene expression analysis

RNA was extracted from tumor specimens and from 2×2×2 mm^3^ tumor xenograft samples (derived from different passages ranging from 1 to 3), which were taken from sacrificed animals. Samples were snap-frozen and stored in liquid nitrogen until use. Total RNA of homogenized tumor samples was prepared with Trizol RNA extraction reagent (Invitrogen) followed by purification using the RNeasy Mini Kit (Qiagen) according to the manufacturer's recommendations. A DNase I (Qiagen) digestion step was included to eliminate genomic DNA. The quality of the total RNA was checked for integrity using RNA LabChips on the Agilent Bioanalyzer 2100 (Agilent Technologies) and the concentration was measured on the Peqlab NanoDrop. Only RNA with a RNA integrity number larger than 6.5 was used for cDNA synthesis. The one-cycle eukaryotic target labeling assay from Affymetrix was used according to manufacturer's instructions and as previously described [36]. Data analysis was performed as previously described [36].

### DNA extraction and mutation analysis

DNA was extracted from paraffin-embedded samples of colon carcinoma with lymphatic and hepatic metastases. For every tumor tissue, 10-µm sections were prepared, and an additional representative 2-µm section was deparaffinised, stained with haematoxylin and eosin, and analysed for detailed morphology. Regions of tumor tissue were marked, and this tissue was extracted with 0.2 M sodium hydroxide in 1 mM edetic acid and neutralised with 100 mM TRIS-TE (pH 6.5). After extraction, DNA was purified with Qiagen PCR purification kit (Qiagen, Hilden, Germany). *KRAS* gene in exon 1 was analysed at codons 12 and 13 with pyrosequencing using a previously described assay which has been shown to be of greater sensitivity [37]. *BRAF* gene in exon 15 at codon 600 and *PIK3CA* gene in exon 9 at codons 539, 542, 545 and 546 and exon 20 at codons 1043, 1044, 1047 and 1049 were analysed with pyrosequencing as previously described [14].

### qRT-PCR

Total RNA was extracted from frozen tissues using TRIzol reagent according to the protocol provided by the manufacturer (Invitrogen, Carlsbad, CA). Total RNA was reverse-transcribed into single-strand complementary DNA (cDNA) using moloney-murine leukemia virus (M-MLV) reverse transcriptase (Promega, Madison, WI). Briefly, the RNA was denatured by heating for 5 min at 70°C, followed by rapid cooling on ice, and then used for reverse transcription (2 µg of total RNA, 25 U of RNase inhibitor, 0.5 mmol/L of each dNTP, 1.5 µmol/L reverse primer and 200 U of M-MLV reverse transcriptase in a total volume of 25 µl). For reverse transcription, tubes were incubated at 42°C for 60 min. The expression of a randomly selected set of genes, including dehydropyrimidine deshydrogenase (*DPD*), nucleotide excision repair-1 (*ERCC-1*), and thymidylate synthase (*TS*) in tissues in original tumors (G0) and tumors from the third generation (G3) was analyzed using a fluorescence-based real-time detection method (ABI PRISM 7700 Sequence Detection System [TaqMan]; Perkin-Elmer Applied Biosystems) as previously described [38], [39]. Specific primer pairs and probes are listed in **Table 1**. The *β*-actin gene was used as an endogenous control for normalization. The qRT-PCR reaction was carried out in triplicate for each sample. The 25 µl PCR mixture was consisted of 1 µl of cDNA template, 1 µl each of sense and anti-sense primers, 0.75 µl of 5′ FAM- and 3′ TAMARA-labeled oligonucleotide probe, 2 µl of dNTP mixture, 5 µl of 5× reaction buffer, and 0.125 µl of Taq DNA polymerase. Cycling conditions were 50°C for 2 minutes, and 95°C for 10 minutes, followed by 46 cycles at 95°C for 15 seconds and 60°C for 1 minute.

**Table 1:** Sequences of primers and probes for qRT-PCR.

Primer and probe sequences	
**DPD**	
Forward primer	5′- AGGACGCAAGGAGGGTTTG -3′
Reverse primer	5′- GTCCGCCGAGTCCTTACTGA -3′
Probe	6FAM-5′- CAGTGCCTACAGTCTCGAGTCTGCCAGTG -3′-TAMRA
**ERCC1**	
Forward primer	5′-GGGAATTTGGCGACGTAATTC-3′
Reverse primer	5′-GCGGAGGCTGAGGAACAG-3′
Probe	6FAM-5′- CACAGGTGCTCTGGCCCAGCACATA -3′-TAMRA
**TS**	
Forward primer	5′-GCCTCGGTGTGCCTTTCA-3′
Reverse primer	5′-GGCTCGATGTGATTCAGGTAAATAT-3′
Probe	6FAM-5′-CACGGGCCTGAAGCCAGGTGACTTTATA-3′-TAMRA
***β*** **-actin**	
Forward primer	5′-TGAGCGCGGCTACAGCTT-3′
Reverse primer	5′-TCCTTAATGTCACGCACGATTT-3′
Probe	6FAM-5′-ACCACCACGGCCGAGCGG-3′-TAMRA

### Western blotting

Protein expression profiles were analyzed by western blotting as previously described [31], [40], [41]. Briefly, lysates for immunoblotting were prepared by adding lysis buffer [50 mM Tris-HCl (pH 7.4), 1% Nonidet P-40, 0.5% sodium deoxycholate, 150 mM NaCl, 0.02% sodium azide, and 0.1% SDS] containing protease and phosphatase inhibitors (Sigma, St. Louis, MO) to the tumor tissue homogenized in fluid nitrogen. After centrifugation at 15,000 rpm at 4°C for 10 min, the supernatants were collected, and the protein concentration was determined using Bio-Rad Protein Assay Kit (Bio-Rad, Hercules, CA). Protein extracts of tumor lysates (30 µg) were added to a loading buffer [10 mmol/L Tris-HCl (pH 6.8), 1% SDS, 25% glycerol, 0.1 mmol/L mercaptoethanol, and 0.03% bromophenol blue], boiled, and separated on 8% to 12% (w/v) polyacrylamide gels in the presence of SDS. Molecular weights of the immunoreactive proteins were estimated based on the relative migration with colored molecular weight protein markers (Amersham Pharmacia Biotech, Piscataway, NJ). After electrophoresis, the protein blots were electro-transferred to PVDF membranes (Millipore, Billerica, MA). Then, the membranes were blocked at room temperature with 5% nonfat milk in TBS [10 mmol/L Tris-HCl (pH 7.5), 0.5 mol/L NaCl, and 0.05% (v/v) Tween 20] buffer for 1 h. The primary antibodies were diluted at 1∶1,000 and the membranes were incubated with primary antibodies overnight at 4°C. The antibodies tested were anti-Akt, anti-ERK, anti-MAPK, anti-mTOR antibodies, anti-EGFR, anti-VEGF, anti-cleaved-caspase-3, anti-PCNA, and phosphorylation-specific antibodies against Akt (Ser^308^ and Ser^473^), ERK (Thr^202^/Tyr^204^), MAPK (Thr^180^/Tyr^182^) and mTOR (Ser^2448^). The next day, the membranes were washed and incubated for 1 h at room temperature with rabbit immunoglobulin G-horseradish peroxidase-conjugated secondary antibodies (Santa Cruz Biotechnology), at a final dilution of 1∶5,000. After washing thrice with TBS, antibody binding was visualized using enhanced chemiluminescence detection system (SuperSignal West Pico, Pierce) as described by the manufacturer and autoradiography. To show equal protein loading, the blots were stripped and reprobed for GAPDH.

### Statistical analysis

Hierarchical clustering of all microarray experiments was done based on all probe sets (54,675 probe sets) represented on the HGU133Plus2.0 array (Affymetrix) with a quality *p*<0.04 using positive correlation and complete linkage. Gene expression of primary tumors was compared with the median arrays of replicate tumors from each xenograft model in a paired *t* test. Drug sensitivity data are presented as mean ± SEM and analyzed by SPSS 16.0 software. Difference among mean of the groups is determined with one-way ANOVA. Comparison is considered to be statistically significant if *p*<0.05.

## Results

### Patient-derived tumor tissues of colon carcinoma and its corresponding lymphatic and hepatic metastases can be implanted efficiently into nude mice

To test whether patient-derived tumor tissues of primary colon carcinoma and its corresponding lymphatic and hepatic metastases can be engrafted in nude mice, we implanted small pieces of freshly tumor tissue into female athymic nude mice subcutaneously. After two to five months, tumors began to appear at the site of implantation with 1000 to 1500 mm^3^ in volume, and xenografts were harvested for serial transplantation. The tumor-bearing rate and tumor growth rate of different generations of PDTT xenografts of primary colon carcinoma and its corresponding lymphatic and hepatic metastases were illustrated in **Table 2**.

**Table 2:** Tumor-bearing rate and tumor growth rate of the PDTT xenograft models.

Tumor-bearing rate			
	G1 (n = 20)	G2 (n = 20)	G3 (n = 20)
Primary colon carcinoma	60%	80%	100%
Colon carcinoma lymphatic metastasis	60%	80%	100%
Colon carcinoma hepatic metastasis	80%	100%	100%

Tumor growth rate a			
	G1 (n = 10)	G2 (n = 10)	G3 (n = 10)
Primary colon carcinoma	141±22.3 ds	93±11.0 ds	34±8.6 ds
Colon carcinoma lymphatic metastasis	111±23.6 ds	67±10.3 ds	30±7.2 ds
Colon carcinoma hepatic metastasis	83±13.2ds	46±8.1 ds	25±6.5 ds

Note:

a measured when tumor volume arrived 1000 mm^3^; G1, the first generation of xenograft; G2, the second generation of xenograft; G3, the third generation of xenograft; ds, days.

### Histological and molecular characterization of xenografts and comparison with original tumors

**Figure 1:**
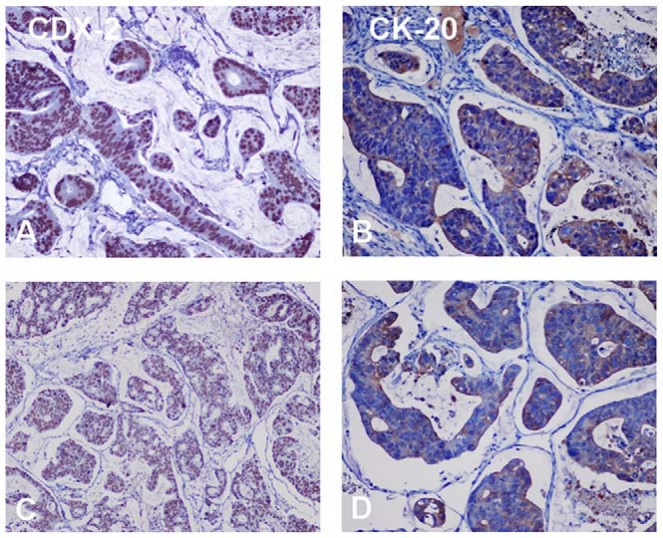
Expression of CDX-2 and CK-20 in the tumor tissues of lymphatic (A and B) and hepatic metastasis (C and D). Original magnifications, ×200.

**Figure 2:**
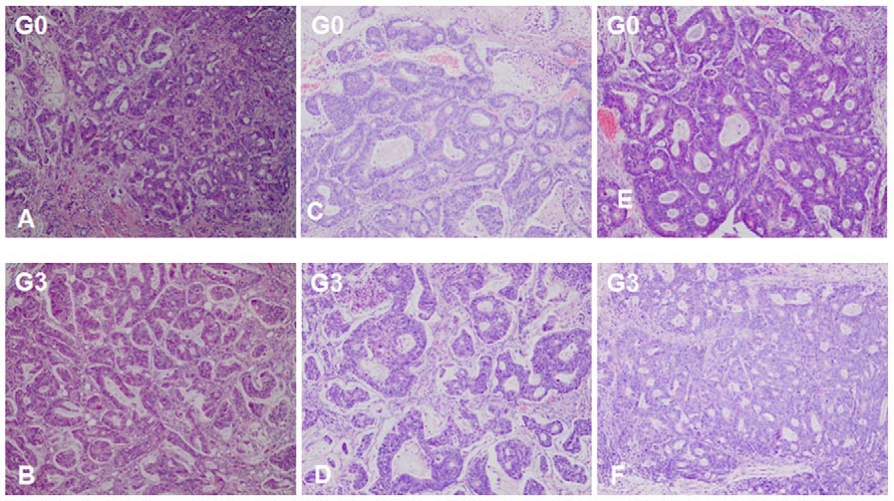
Representative H&E stained tissues of primary colon carcinoma (A and B) and its corresponding lymphatic (C and D) and hepatic (E and F) metastases and their early-generation of xenograft tumors. G0, the primary tumors; G3, the third generation of xenografts. Original magnifications, ×100.

**Figure 3:**
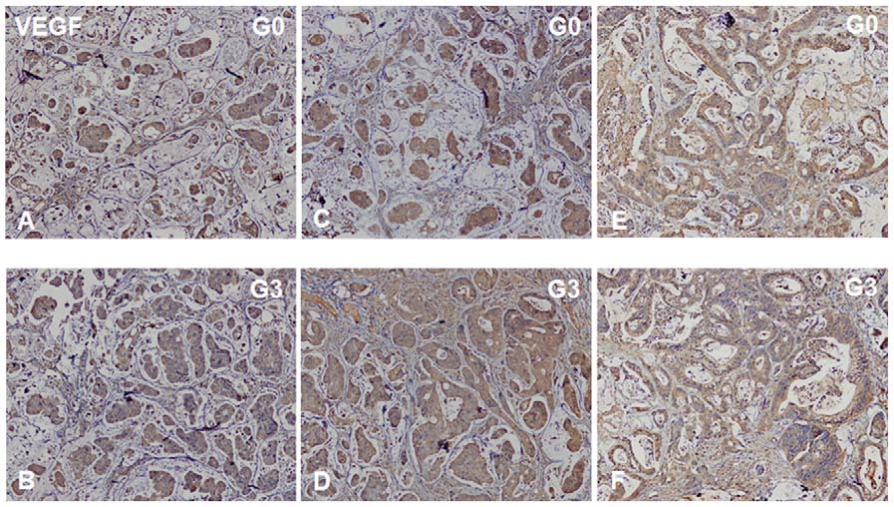
VEGF immunohistochemical staining of primary tumor tissues of primary colon carcinoma (A and B) and its corresponding lymphatic (C and D) and hepatic (E and F) metastases and their early-generation xenograft tumor tissues. G0, the primary tumors; G3, the third generation of xenografts. Original magnifications, ×100.

Using PDTT xenografts as models for preclinical anticancer drug development is based on the assumption that the xenografts would closely resemble the corresponding original tumors. For this purpose, H&E staining, immunohistochemical staining, genome-wide gene expression analysis, pyrosequencing, qRT-PCR and western blotting were used to determine the biological stability of the xenograft during serial transplantation compared with the original tumor tissues. In this study, CK-20 and CDX-2 were used as markers for determining the lymphatic and hepatic metastases with a colon carcinoma primary. Immunohistochemical staining showed a positive expression of CK-20 and CDX-2 in the metastases (**Fig. 1**), which ascertained lymphatic and hepatic metastases with an epithelial origin. Histological examination of the H&E sections showed that the PDTT xenografts were adenocarcinoma with features similar to the original surgical specimens. **Fig. 2** shows the morphology of the original tumors (G0) of primary colon carcinoma with lymphatic and hepatic metastases and their third generation (G3) implants in nude mice. There were no significant morphological differences between the tumor resected from the patient and the initial successful implants. The explanted tumor pieces also showed similar VEGF and EGFR expressions compared to the corresponding original tumors (**Fig. 3** **,** **4**).

**Figure 4:**
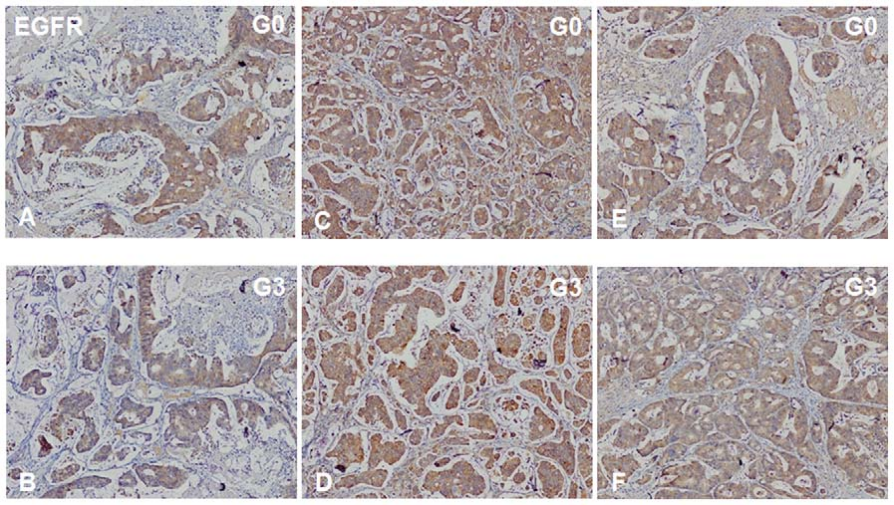
EGFR immunohistochemical staining of primary tumor tissues of primary colon carcinoma (A and B) and its corresponding lymphatic (C and D) and hepatic (E and F) metastases and their early-generation xenograft tumor tissues. G0, the primary tumors; G3, the third generation of xenografts. Original magnifications, ×100.

**Figure 5:**
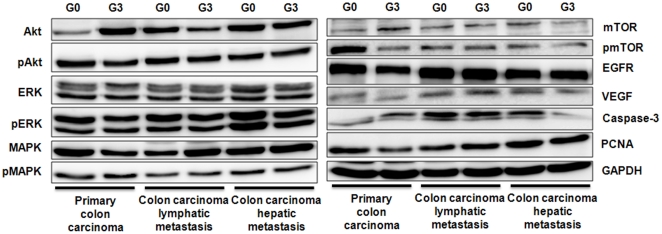
Exemplified immunoblotting data of the proteins Akt, pAkt (Ser^308^ and Ser^473^), ERK, pERK (Thr^202^/Tyr^204^), MAPK, pMAPK (Thr^180^/Tyr^182^), mTOR, pmTOR (Ser^2448^), EGFR, VEGF, Casepase-3, PCNA and GAPDH (as loading control) of primary colon carcinoma and its corresponding lymphatic and hepatic metastases and their early-generation xenograft tumor tissues. G0, the primary tumors; G3, the third generation of xenografts.

**Figure 6:**
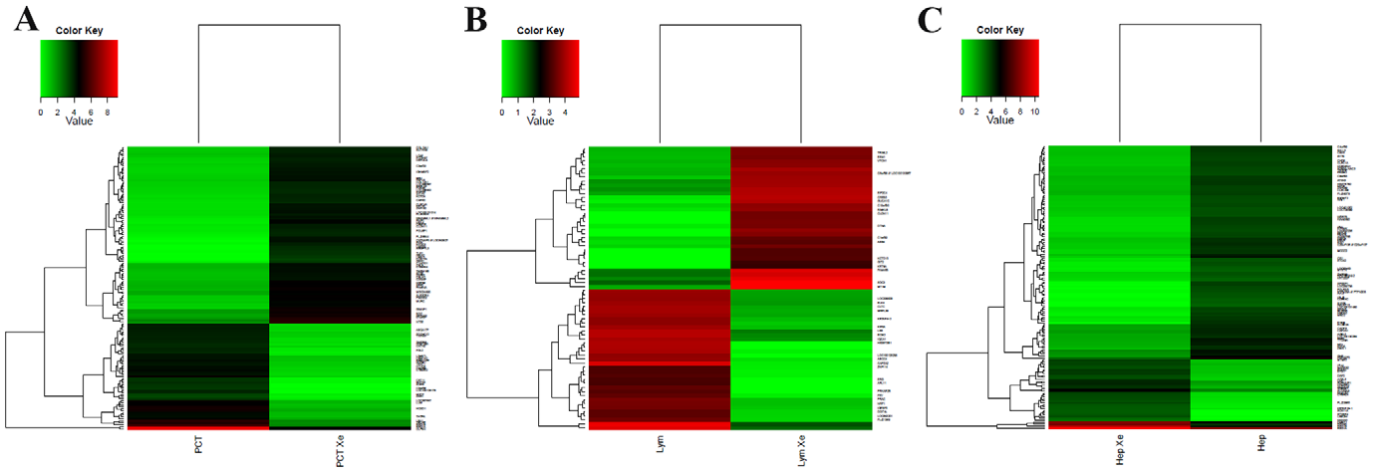
Gene expression profiling of patient-derived tumor tissues and corresponding xenografts. A, hierarchical clustering based on 140 probe sets differentially expressed between primary colon carcinoma and its xenograft. B, hierarchical clustering based on 70 probe sets differentially expressed between colon carcinoma lymphatic metastasis and its xenograft. C, hierarchical clustering based on 145 probe sets differentially expressed between colon carcinoma hepatic metastasis and its xenograft. PCT, primary colon carcinoma. PCT Xe, primary colon carcinoma xenograft. Lym, colon carcinoma lymphatic metastasis. Lym Xe, colon carcinoma lymphatic metastasis xenograft. Hep, colon carcinoma hepatic metastasis. Hep Xe, colon carcinoma hepatic metastasis xenograft.

When using the PDTT xenograft models for testing of the anticancer agents, especially molecularly targeted agents, detailed characterization of the molecules is indispensable. For this purpose, GeneChip HGU133Plus2.0 expression arrays (Affymetrix) was used to determine the status of the genome-wide gene expression of the xenograft models, pyrosequencing was used to determine the mutation status of some selected genes of the xenograft models, qRT-PCR was used to determine the status of some randomized selected genes of the xenograft models, western blotting was used to determine the expression of some randomized selected signaling pathway proteins of the xenograft models during serial transplantation compared with the original tumor tissues. Our results showed that early passages of the PDTT xenograft models of primary colon carcinoma with lymphatic and hepatic metastases revealed a high degree of similarity with the original clinical tumor samples with regard to *KRAS*, *BRAF*, *EGFR*, and *PIK3CA* mutation status (**see Fig. S1, S2, S3, S4**) as well as mRNA expression (data not shown). With regards to the western blotting analysis of ‘randomized selected signaling proteins’, early passages of the PDTT xenograft models of colon carcinoma lymphatic and hepatic metastases revealed a high degree of similarity with the original clinical tumor samples though these phenomena did not exist between original clinical tumor sample of primary colon cancer and its xenografts (**Fig. 5**). Based on the results of genome-wide gene expression analysis, we calculated the correlation coefficient between primary cancer and the xenograft derived thereof. The correlation coefficient ranged between 0.988 and 0.991 (0.991 between primary colon carcinoma and its xenograft, 0.989 between lymphatic metastasis and its xenograft, and 0.988 between hepatic metastasis and its xenograft) indicating a high degree of similarity between the primary cancer and the corresponding xenograft model. A paired *t* test between the primary tumors and xenografts revealed limited differentially expressed probe sets. Clustering based on these probe sets showed a limited distinction between primary tumors and xenografts (**Table S1, S2, S3,** **Fig. 6**).

### PDTT xenograft models of colon carcinoma and its corresponding lymphatic and hepatic metastases for assessment of a novel VEGF targeted agent

Our results indicated that during sequential passage, the PDTT xenograft models retained their similarity to the corresponding original tumors in morphology, architecture and molecular signatures thus could be used for rapid screening of potential therapeutics. To improve confidence of a novel VEGF blocker during its early preclinical studies allowing for a faster decision is one of purposes of using these xenograft models. To explore the value of FP3 as an antitumor therapeutic and to compare it to other effective agents targeting the VEGF pathway, we evaluated its ability to block the growth of these xenograft models.

**Figure 7:**
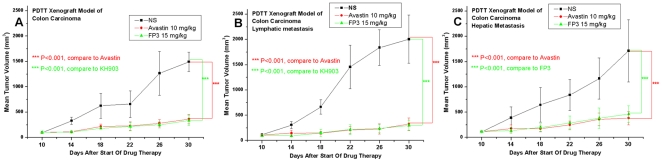
Response curve of FP3 and Avastin in the PDTT xenograft models of primary colon carcinoma (A), lymphatic metastasis (B), and hepatic metastasis (C). Ten mice per group were treated with the corresponding agent according to Materials and Methods. Data shown are means ± SEM. The differences between control tumor volumes, FP3-treated, and Avastin-treated tumor volumes were analyzed by using one-way ANOVA. ^***^ *p*<0.001, versus control. Experiments were repeated at least two times with similar results.

After implantation, tumors were allowed to grow for 10 days, forming large retroperitoneal tumors >100 mm^3^. Injections of FP3 (15 mg/kg body weight), Avastin (10 mg/kg body weight) or saline were then given i.v. twice per week for 21 days, after which the animals were killed and tumors excised and measured. No body weight-related toxicity was found in each group. FP3 significantly inhibited the growth of xenografts of primary colon carcinoma and its lymphatic and hepatic metastases in nude mice (**Fig. 7**), and resembled the well-defined and generally accepted antitumor activity of Avastin [42].

**Figure 8:**
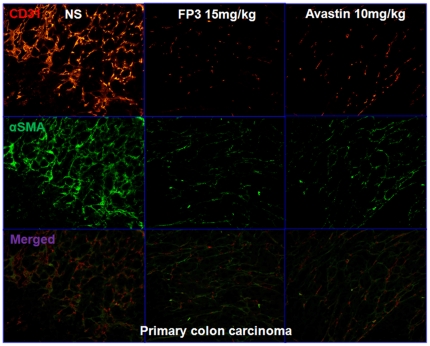
FP3 decreased vascular structure in the xenograft model of primary colon carcinoma. Vasculature was examined by angiography with immunostaining for endothelial cells (using anti-CD31 antibody; bar = 100 µm), and pericytes (using anti-α-SMA antibody; bar = 100 µm). There was a paucity of vessels identified in FP3-treated tumors.

**Figure 9:**
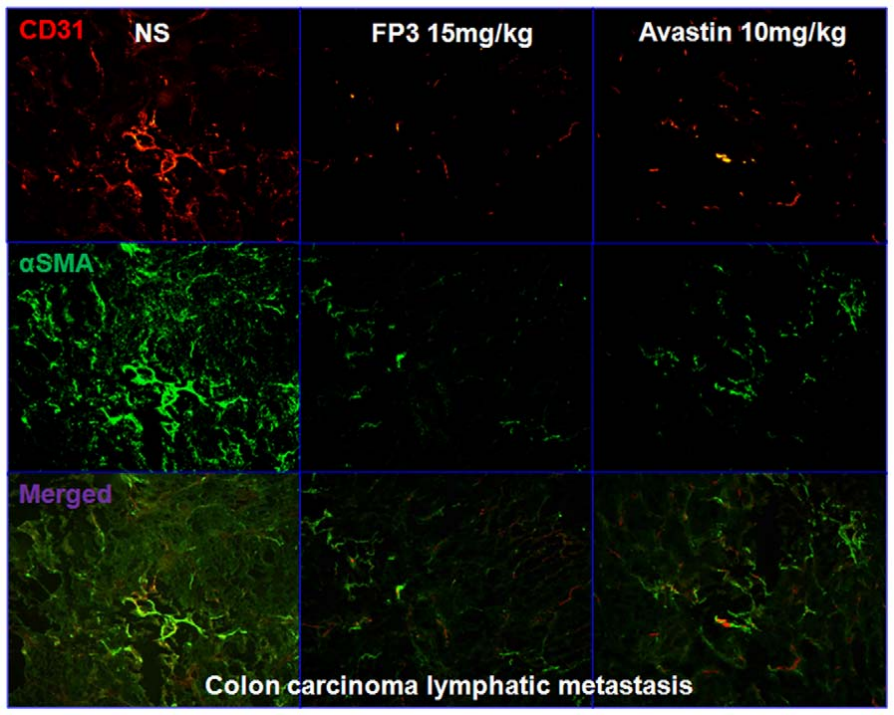
FP3 decreased vascular structure in the xenograft model of colon carcinoma lymphatic metastasis. Vasculature was examined by angiography with immunostaining for endothelial cells (using anti-CD31 antibody; bar = 100 µm), and pericytes (using anti-α-SMA antibody; bar = 100 µm). There was a paucity of vessels identified in FP3-treated tumors.

**Figure 10:**
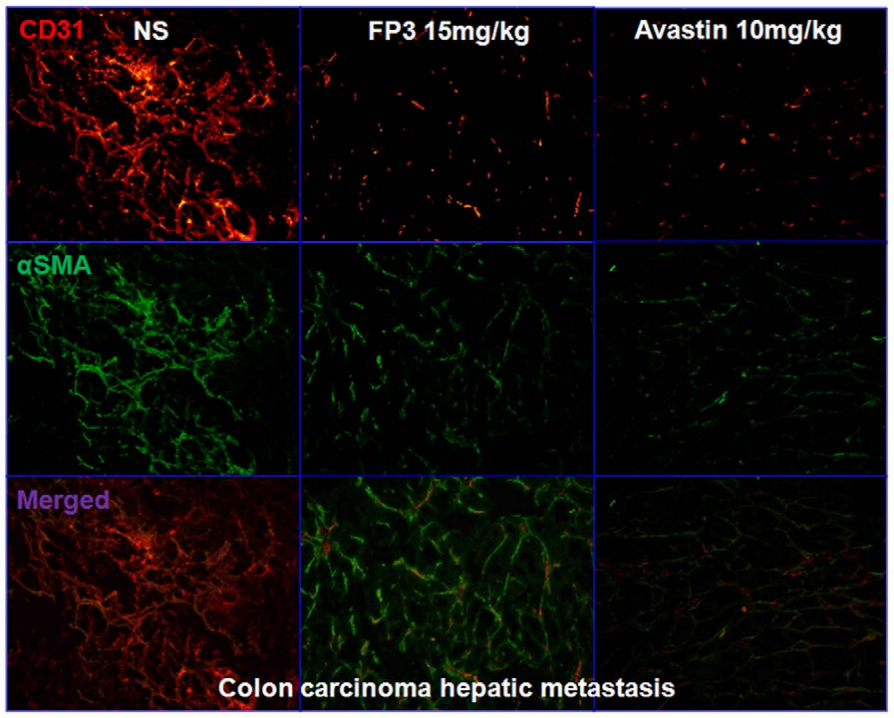
FP3 decreased vascular structure in the xenograft model of colon carcinoma hepatic metastasis. Vasculature was examined by angiography with immunostaining for endothelial cells (using anti-CD31 antibody; bar = 100 µm), and pericytes (using anti-α-SMA antibody; bar = 100 µm). There was a paucity of vessels identified in FP3-treated tumors.

To evaluate the effects of FP3 on tumor-associated angiogenesis, selected tumors from the above studies were sectioned and immunostained with antibodies to CD31 and α-SMA, so that the vasculature could be visualized (**Fig. 8** **,** **9** **,** **10**). This analysis revealed that vasculature was nearly absent in FP3-treated xenografts. FP3 (treatment for 21 days) almost completely blocked tumor-associated angiogenesis, with the stunted tumors being largely avascular (**Fig. 8: B, E, and H** **;** **Fig. 9: B, E, and H** **;** **Fig. 10: B, E, and H**). In contrast to the FP3-treated tumors, control tumors in saline-treated mice not only were much larger but also had a very high vascular density (**Fig. 8: A, D, and G** **;** **Fig. 9: A, D, and G** **;** **Fig. 10: A, D, and G**). These results indicate that FP3 administration reduces xenograft size and concurrently causes decreased microvessel growth.

**Figure 11:**
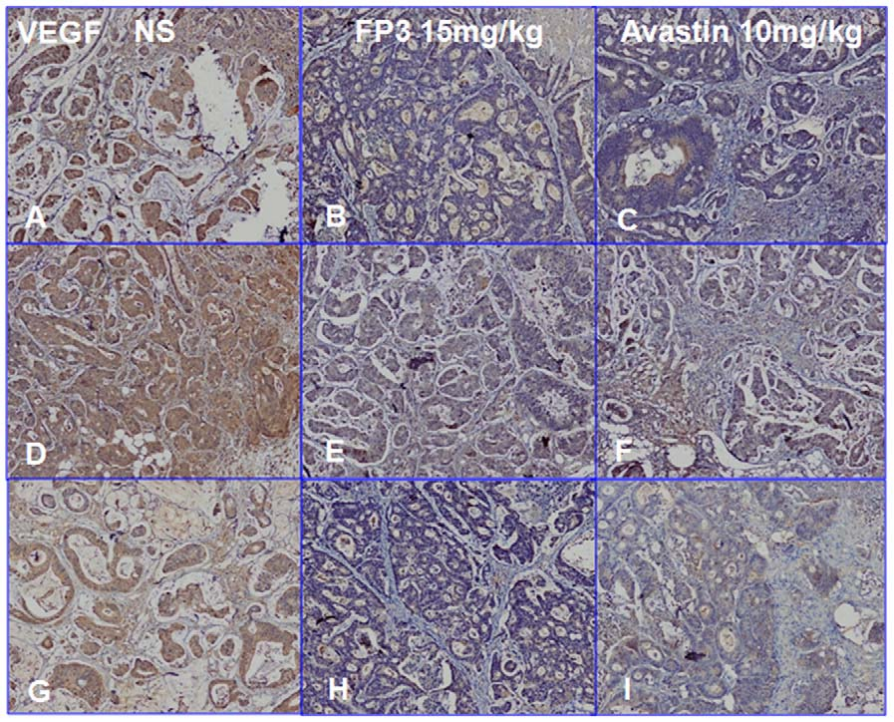
Effects of FP3 and Avastin on the expression of VEGF in the PDTT xenograft models of primary colon carcinoma (A–C), lymphatic metastasis (D–F), and hepatic metastasis (G–I). Original magnifications, ×100.

**Figure 12:**
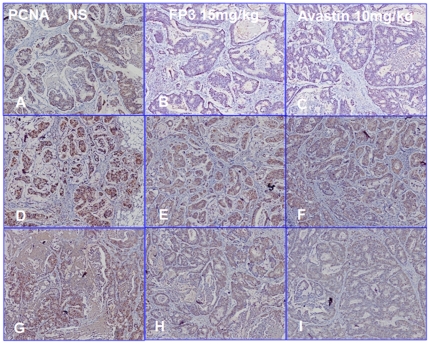
Effects of FP3 and Avastin on the expression of PCNA in the PDTT xenograft models of primary colon carcinoma (A–C), lymphatic metastasis (D–F), and hepatic metastasis (G–I). Original magnifications, ×100.

**Figure 13:**
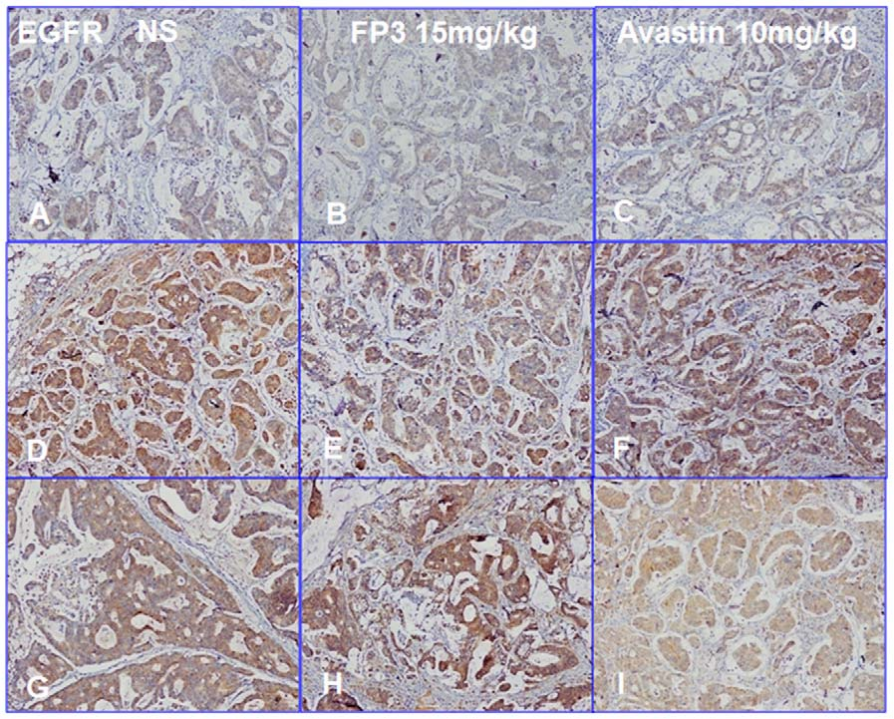
Effects of FP3 and Avastin on the expression of EGFR in the PDTT xenograft models of primary colon carcinoma (A–C), lymphatic metastasis (D–F), and hepatic metastasis (G–I). Original magnifications, ×100.

We next measured cell proliferation in the treated tumors. By immunohistochemical staining, we found that VEGF expression (**Fig. 11**) and PCNA expression (**Fig. 12**) in FP3- and Avastin-treated tumors were significantly suppressed. However, EGFR expression was not significantly different between any of the treatment groups and saline-treated controls (**Fig. 13**), suggesting that levels of EGFR are unlikely to be altered by the treatments.

**Figure 14:**
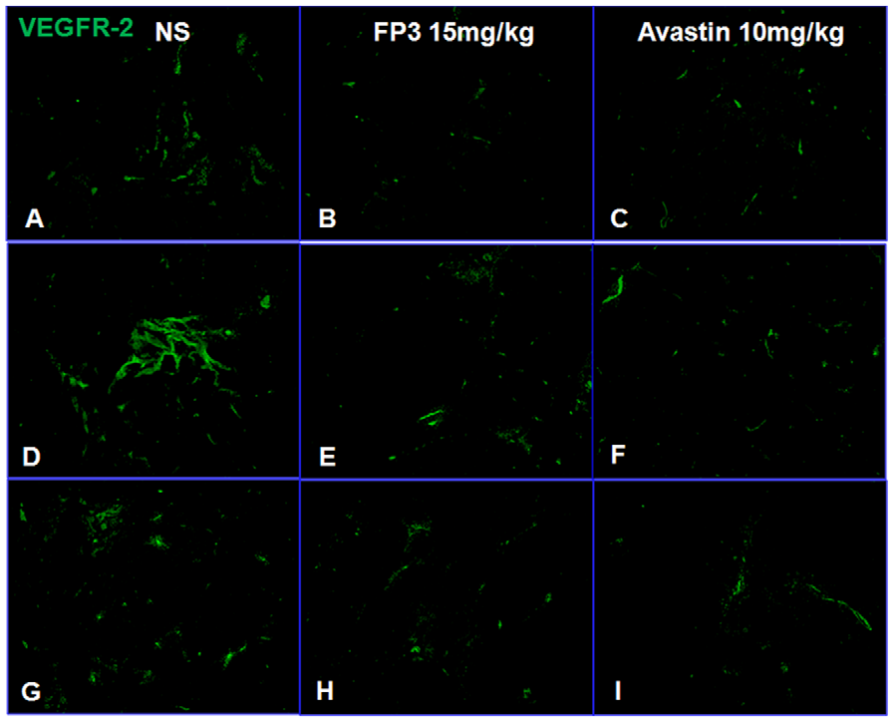
Effects of FP3 and Avastin on the expression of VEGFR-2 in the PDTT xenograft models of primary colon carcinoma (A–C), lymphatic metastasis (D–F), and hepatic metastasis (G–I). Original magnifications, ×100.

Because inhibition of VEGF signaling can decrease VEGFR-2 expression in certain types of blood vessels [43], [44], we asked whether VEGF/VEGFR blockade decreased receptor expression in our tumor models. Treatment of the xenograft models with FP3 for 21 days, decreased expression of VEGFR-2, a marker for growing vasculature (**Fig. 14** **,** **15**). These results were consistent with the disappearance of endothelial cells expressing this receptor (**Fig. 8** **,** **9** **,** **10**).

**Figure 15:**
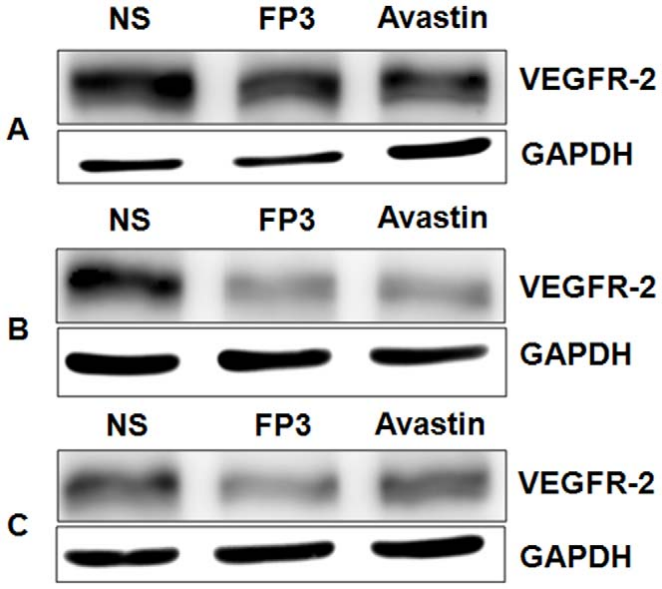
Western blotting analysis showing the effects of FP3 and Avastin on the expression of VEGFR-2 protein in the PDTT xenograft models of primary colon carcinoma (A), lymphatic metastasis (B), and hepatic metastasis (C).

## Discussion

Rodent tumor models currently being used and which include transgenic tumor models, and those generated by planting human tumor cell lines subcutaneously in immunodeficient mice, do not sufficiently represent clinical cancer characteristics, especially with regard to metastasis and drug sensitivity [29]. The increasingly used PDTT xenografts models implanted subcutaneously or in subrenal capsule in immunodeficient mice provide a more accurate reflection of human tumor biological characteristics than tumor cell lines. The ability to passage patients' fresh tumor tissues into large numbers of immunodeficient mice provides possibilities for better preclinical testing of new therapies for the treatment and better outcome for cancer.

In our study, we successfully established the PDTT xenograft models of primary colon carcinoma and its corresponding lymphatic and hepatic metastases. One of vital questions relating to PDTT xenografts is whether tumor passage in the experimental mice alters the phenotype of primary cancer cells. When developing the anticancer agents, especially molecularly targeted agents, detailed characterisation of the molecules is indispensable. If there are fundamental changes in tumors from before to after their engrafting, the model may not do well in reflecting the features of human cancers. Therefore, the practicability of this model as a screening platform for new drug development for this disease will be limited. Similarly, the value of this model as a tool to individualise patient treatment would be limited [40]. Using PDTT xenografts as models for preclinical anticancer drug development is based on the assumption that the xenografts would closely resemble the corresponding original tumors. In our study, we firstly demonstrated that early passages of the PDTT xenograft models of primary colon carcinoma, lymphatic and hepatic metastases revealed a high degree of similarity with the original clinical tumor sample with regard to histology (**Fig. 2**), immunohistochemistry (**Fig. 3** **,** **4**), mutation status (**see Fig. S1, S2, S3, S4**), mRNA expression (data not shown) as well as genes expression (**Fig. 6**). As these xenografts models retain similar histopathological features and molecular signatures as the original tumors, they could be used for rapid screening of potential therapeutics not only on the primary tumor, but also on its corresponding metastases.

One of the general applications of the PDTT xenograft models of primary colon carcinoma and its corresponding lymphatic and hepatic metastases is that they can be used as an in vivo screening tool to test the response of both the primary tumor and the metastases to some certain novel drugs. Furthermore, these models may help us to clear whether the primary tumors and corresponding metastases have different response to the same anticancer drugs. In this study, the PDTT xenograft models of primary colon carcinoma and its corresponding lymphatic and hepatic metastases were used for assessment of a novel VEGF targeted agent, FP3. As shown in results, FP3 significantly inhibited the growth of xenografts of primary colon carcinoma and its corresponding lymphatic and hepatic metastases and concurrently caused inhibition of tumor vessels growth dramatically [**Fig. 7** **,** **8** **,** **9** **,** **10**]. These results coincide with our previous works [24], [28].

In conclusion, our work successfully established the PDTT xenograft models of colon carcinoma with lymphatic and hepatic metastasis useful for testing of a novel molecularly anti-VEGF targeted agent, FP3. Early passages of the PDTT xenograft models of primary colon carcinoma, lymphatic and hepatic metastases revealed a high degree of similarity with the original clinical tumor samples. They provide appropriate models for testing of novel molecularly targeted agents. These models may also help us to clear whether the primary tumors and corresponding metastases have different response to the same anticancer drugs.

## Supporting Information

Figure S1 **Mutation analysis by pyrosequencing.** Sequence data of *KRAS* gene in exon 1 at codons 12 and 13 of primary tumor tissues and their early-generation xenograft models tumor tissues. A, standard wild-type sample. Tumor tissues from surgical specimens of primary colon carcinoma (B) and its corresponding lymphatic metastasis (D) and hepatic metastasis (F). Tumor tissues from the third generation of xenograft models of primary colon carcinoma (C) and its corresponding lymphatic metastasis (E) and hepatic metastasis (G).
(TIF)

Figure S2 **Mutation analysis by pyrosequencing.** Sequence data of *BRAF* gene in exon 15 at codon 600 of primary tumor tissues and their early-generation xenograft models tumor tissues. A, standard wild-type sample. Tumor tissues from surgical specimens of primary colon carcinoma (B) and its corresponding lymphatic metastasis (D) and hepatic metastasis (F). Tumor tissues from the third generation of xenograft models of primary colon carcinoma (C) and its corresponding lymphatic metastasis (E) and hepatic metastasis (G).
(TIF)

Figure S3 **Mutation analysis by pyrosequencing.** Sequence data of *PIK3CA* gene in exon 9 at codons 539, 542, 545 and 546 of primary tumor tissues and their early-generation xenograft models tumor tissues. A, standard wild-type sample. Tumor tissues from surgical specimens of primary colon carcinoma (B) and its corresponding lymphatic metastasis (D) and hepatic metastasis (F). Tumor tissues from the third generation of xenograft models of primary colon carcinoma (C) and its corresponding lymphatic metastasis (E) and hepatic metastasis (G).
(TIF)

Figure S4 **Mutation analysis by pyrosequencing.** Sequence data of *PIK3CA* gene in exon 20 at codons 1043, 1044, 1047 and 1049 of primary tumor tissues and their early-generation xenograft models tumor tissues. A, standard wild-type sample. Tumor tissues from surgical specimens of primary colon carcinoma (B) and its corresponding lymphatic metastasis (D) and hepatic metastasis (F). Tumor tissues from the third generation of xenograft models of primary colon carcinoma (C) and its corresponding lymphatic metastasis (E) and hepatic metastasis (G).
(TIF)

Table S1 **Genes differentially expressed in patient primary colon cancer specimens and its xenograft.**
(DOC)

Table S2 **Genes differentially expressed in patient colon cancer lymphatic metastasis specimens and its xenograft.**
(DOC)

Table S3 **Genes differentially expressed in patient colon cancer hepatic metastasis specimens and its xenograft.**
(DOC)

## References

[1] Morton CL, Houghton PJ (2007). Establishment of human tumor xenografts in immunodeficient mice.. Nat Protoc.

[2] Voskoglou-Nomikos T, Pater JL, Seymour L (2003). Clinical predictive value of the in vitro cell line, human xenograft, and mouse allograft preclinical cancer models.. Clin Cancer Res.

[3] Johnson JI, Decker S, Zaharevitz D, Rubinstein LV, Venditti JM (2001). Relationships between drug activity in NCI preclinical in vitro and in vivo models and early clinical trials.. Br J Cancer.

[4] Sausville EA, Burger AM (2006). Contributions of human tumor xenografts to anticancer drug development.. Cancer Res.

[5] Jin K, He K, Teng F, Han N, Li G (2011). Heterogeneity in primary tumors and corresponding metastases: could it provide us with any hints to personalize cancer therapy?. Pers Med.

[6] Gong Y, Booser DJ, Sneige N (2005). Comparison of HER-2 status determined by fluorescence in situ hybridization in primary and metastatic breast carcinoma.. Cancer.

[7] Gancberg D, Di Leo A, Cardoso F, Rouas G, Pedrocchi M (2002). Comparison of HER-2 status between primary breast cancer and corresponding distant metastatic sites.. Ann Oncol.

[8] Regitnig P, Schippinger W, Lindbauer M, Samonigg H, Lax SF (2004). Change of HER-2/neu status in a subset of distant metastases from breast carcinomas.. J Pathol.

[9] Bozzetti C, Personeni N, Nizzoli R, Guazzi A, Flora M (2003). HER-2/neu amplification by fluorescence in situ hybridization in cytologic samples from distant metastatic sites of breast carcinoma.. Cancer.

[10] Tanner M, Järvinen P, Isola J (2001). Amplification of HER-2/neu and topoisomerase IIalpha in primary and metastatic breast cancer.. Cancer Res.

[11] Tapia C, Savic S, Wagner U, Schönegg R, Novotny H (2007). HER2 gene status in primary breast cancers and matched distant metastases.. Breast Cancer Res.

[12] Akcakanat A, Sahin A, Shaye AN, Velasco MA, Meric-Bernstam F (2008). Comparison of Akt/mTOR signaling in primary breast tumors and matched distant metastases.. Cancer.

[13] Wu JM, Fackler MJ, Halushka MK, Molavi DW, Taylor ME (2008). Heterogeneity of breast cancer metastases: comparison of therapeutic target expression and promoter methylation between primary tumors and their multifocal metastases.. Clin Cancer Res.

[14] Baldus SE, Schaefer KL, Engers R, Hartleb D, Stoecklein NH (2010). Prevalence and heterogeneity of KRAS, BRAF, and PIK3CA mutations in primary colorectal adenocarcinomas and their corresponding metastases.. Clin Cancer Res.

[15] Molinari F, Martin V, Saletti P, De Dosso S, Spitale A (2009). Differing deregulation of EGFR and downstream proteins in primary colorectal cancer and related metastatic sites may be clinically relevant.. Br J Cancer.

[16] Scartozzi M, Bearzi I, Berardi R, Mandolesi A, Fabris G (2004). Epidermal growth factor receptor (EGFR) status in primary colorectal tumors does not correlate with EGFR expression in related metastatic sites: implications for treatment with EGFR-targeted monoclonal antibodies.. J Clin Oncol.

[17] Scartozzi M, Bearzi I, Berardi R, Mandolesi A, Pierantoni C (2007). Epidermal growth factor receptor (EGFR) downstream signalling pathway in primary colorectal tumours and related metastatic sites: optimising EGFR-targeted treatment options.. Br J Cancer.

[18] Sasatomi E, Finkelstein SD, Woods JD, Bakker A, Swalsky PA (2002). Comparison of accumulated allele loss between primary tumor and lymph node metastasis in stage II non-small cell lung carcinoma: implications for the timing of lymph node metastasis and prognostic value.. Cancer Res.

[19] Park S, Holmes-Tisch AJ, Cho EY, Shim YM, Kim J (2009). Discordance of molecular biomarkers associated with epidermal growth factor receptor pathway between primary tumors and lymph node metastasis in non-small cell lung cancer.. J Thorac Oncol.

[20] Li Z, Jin K, Lan H, Teng L (2011). Heterogeneity in primary colorectal cancer and its corresponding metastases: a potential reason of EGFR-targeted therapy failure?. Hepatogastroenterology.

[21] Monaco SE, Nikiforova MN, Cieply K, Teot LA, Khalbuss WE (2010). A comparison of EGFR and KRAS status in primary lung carcinoma and matched metastases.. Hum Pathol.

[22] Loupakis F, Pollina L, Stasi I, Ruzzo A, Scartozzi M (2009). PTEN expression and KRAS mutations on primary tumors and metastases in the prediction of benefit from cetuximab plus irinotecan for patients with metastatic colorectal cancer.. J Clin Oncol.

[23] Teng LS, Jin KT, He KF, Wang HH, Cao J (2010). Advances in combination of antiangiogenic agents targeting VEGF-binding and conventional chemotherapy and radiation for cancer treatment.. J Chin Med Assoc.

[24] Jin K, He K, Teng F, Li G, Wang H (2011). FP3: a novel VEGF blocker with antiangiogenic effects in vitro and antitumor effects in vivo.. Clin Transl Oncol.

[25] Zhang M, Zhang J, Yan M, Li H, Yang C (2008). Recombinant anti-vascular endothelial growth factor fusion protein efficiently suppresses choridal neovasularization in monkeys.. Mol Vis.

[26] Zhang M, Yu D, Yang C, Xia Q, Li W (2009). The pharmacology study of a new recombinant human VEGF receptor-fc fusion protein on experimental choroidal neovascularization.. Pharm Res.

[27] Zhang M, Zhang J, Yan M, Luo D, Zhu W (2011). A phase 1 study of KH902, a vascular endothelial growth factor receptor decoy, for exudative age-related macular degeneration.. Ophthalmology.

[28] Jin K, He K, Han N, Li G, Wang H (2011). Establishment of a PDTT xenograft model of gastric carcinoma and its application in personalized therapeutic regimen selection.. Hepatogastroenterology.

[29] Jin K, Teng L, Shen Y, He K, Xu Z (2010). Patient-derived human tumour tissue xenografts in immunodeficient mice: a systematic review.. Clin Transl Oncol.

[30] Jin K, He K, Li G, Teng L (2010). Personalized cancer therapy using a patient-derived tumor tissue xenograft model: a translational field worthy of exploring further?. Pers Med.

[31] Huynh H, Chow PK, Ooi LL, Soo KC (2002). A possible role for insulin-like growth factor-binding protein-3 autocrine/paracrine loops in controlling hepatocellular carcinoma cell proliferation.. Cell Growth Differ.

[32] Wang Y, Xue H, Cutz JC, Bayani J, Mawji NR (2005). An orthotopic metastatic prostate cancer model in SCID mice via grafting of a transplantable human prostate tumor line.. Lab Invest.

[33] Mancuso MR, Davis R, Norberg SM, O'Brien S, Sennino B (2006). Rapid vascular regrowth in tumors after reversal of VEGF inhibition.. J Clin Invest.

[34] Morikawa S, Baluk P, Kaidoh T, Haskell A, Jain RK (2002). Abnormalities in pericytes on blood vessels and endothelial sprouts in tumors.. Am J Pathol.

[35] Baluk P, Morikawa S, Haskell A, Mancuso M, McDonald DM (2003). Abnormalities of basement membrane on blood vessels and endothelial sprouts in tumors.. Am J Pathol.

[36] Fichtner I, Rolff J, Soong R, Hoffmann J, Hammer S (2008). Establishment of patient-derived non-small cell lung cancer xenografts as models for the identification of predictive biomarkers.. Clin Cancer Res.

[37] Ogino S, Kawasaki T, Brahmandam M, Yan L, Cantor M (2005). Sensitive sequencing method for KRAS mutation detection by Pyrosequencing.. J Mol Diagn.

[38] Gibson UE, Heid CA, Williams PM (1996). A novel method for real time quantitative RT-PCR.. Genome Res.

[39] Schneider S, Uchida K, Brabender J, Baldus SE, Yochim J (2005). Downregulation of TS, DPD, ERCC1, GST-Pi, EGFR, and HER2 gene expression after neoadjuvant three-modality treatment in patients with esophageal cancer.. J Am Coll Surg.

[40] Rubio-Viqueira B, Jimeno A, Cusatis G, Zhang X, Iacobuzio-Donahue C (2006). An in vivo platform for translational drug development in pancreatic cancer.. Clin Cancer Res.

[41] Perez-Soler R, Kemp B, Wu QP, Mao L, Gomez J (2000). Response and determinants of sensitivity to paclitaxel in human non-small cell lung cancer tumors heterotransplanted in nude mice.. Clin Cancer Res.

[42] Teng LS, Jin KT, He KF, Wang HH, Cao J (2010). Advances in combination of antiangiogenic agents targeting VEGF-binding and conventional chemotherapy and radiation for cancer treatment.. J Chin Med Assoc.

[43] Huang J, Frischer JS, Serur A, Kadenhe A, Yokoi A (2003). Regression of established tumors and metastases by potent vascular endothelial growth factor blockade.. Proc Natl Acad Sci USA.

[44] Baffert F, Thurston G, Rochon-Duck M, Le T, Brekken R (2004). Age-related changes in VEGF-dependency and angiopoietin-1 induced plasticity of adult blood vessels.. Circ Res.

