# Supplementary figures and images for: Assessment of a Novel VEGF Targeted Agent Using Patient-Derived Tumor Tissue Xenograft Models of Colon Carcinoma with Lymphatic and Hepatic Metastases
Source: PLoS One. 2011 Dec 2;6(12):e28384. doi: 10.1371/journal.pone.0028384 (PMC3229582; doi:10.1371/journal.pone.0028384)

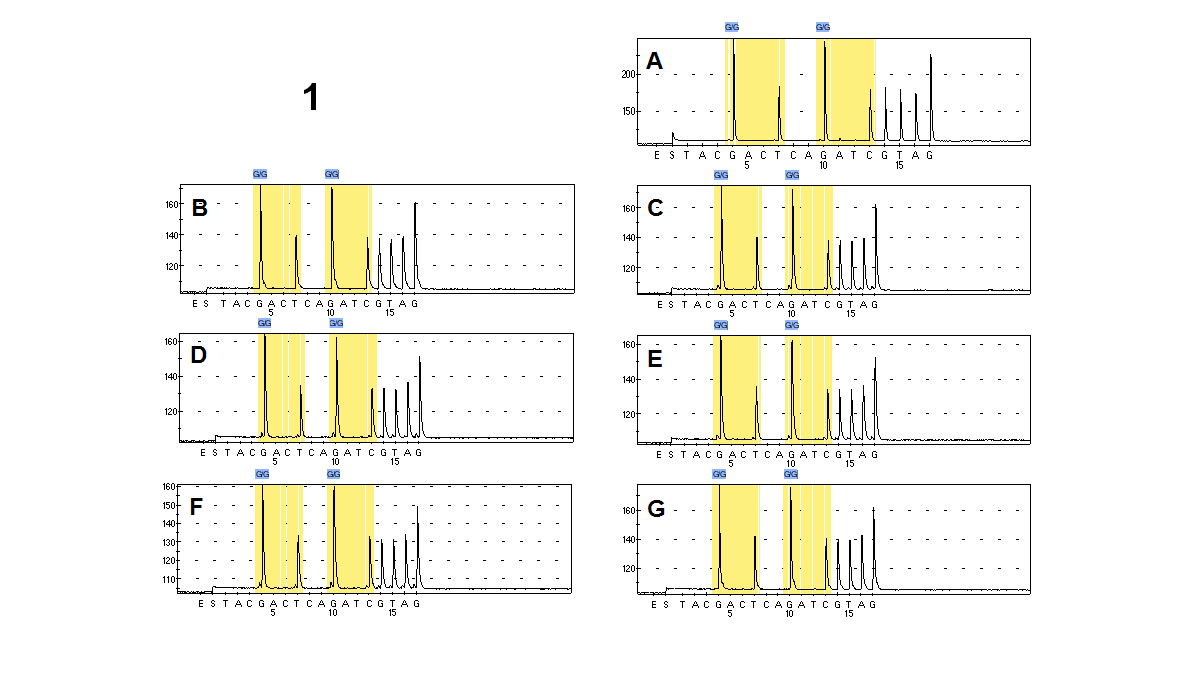

Supplement: Figure S1 — Mutation analysis by pyrosequencing. Sequence data of KRAS gene in exon 1 at codons 12 and 13 of primary tumor tissues and their early-generation xenograft models tumor tissues. A, standard wild-type sample. Tumor tissues from surgical specimens of primary colon carcinoma (B) and its corresponding lymphatic metastasis (D) and hepatic metastasis (F). Tumor tissues from the third generation of xenograft models of primary colon carcinoma (C) and its corresponding lymphatic metastasis (E) and hepatic metastasis (G). (TIF) [file pone.0028384.s001.tif]

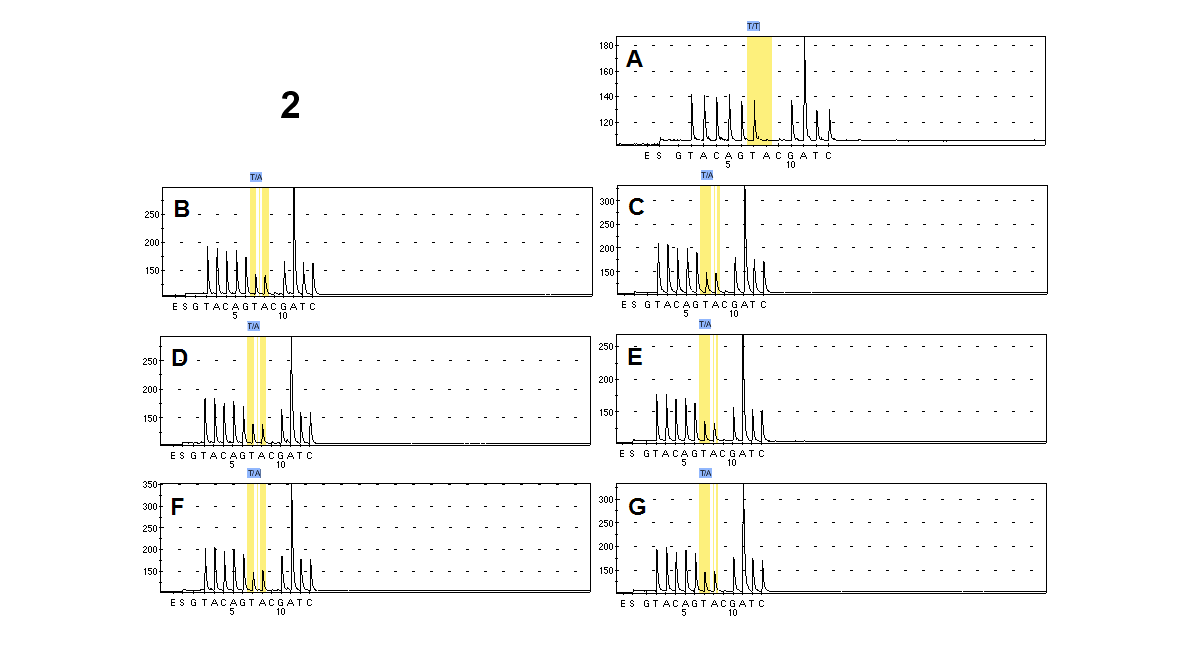

Supplement: Figure S2 — Mutation analysis by pyrosequencing. Sequence data of BRAF gene in exon 15 at codon 600 of primary tumor tissues and their early-generation xenograft models tumor tissues. A, standard wild-type sample. Tumor tissues from surgical specimens of primary colon carcinoma (B) and its corresponding lymphatic metastasis (D) and hepatic metastasis (F). Tumor tissues from the third generation of xenograft models of primary colon carcinoma (C) and its corresponding lymphatic metastasis (E) and hepatic metastasis (G). (TIF) [file pone.0028384.s002.tif]

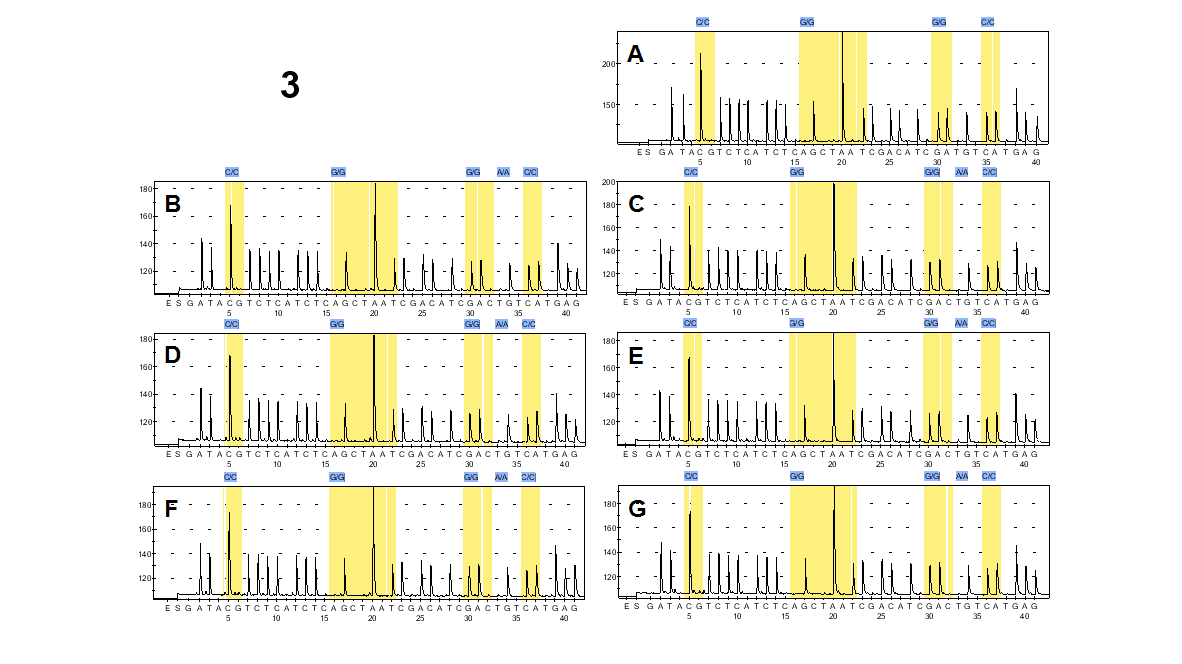

Supplement: Figure S3 — Mutation analysis by pyrosequencing. Sequence data of PIK3CA gene in exon 9 at codons 539, 542, 545 and 546 of primary tumor tissues and their early-generation xenograft models tumor tissues. A, standard wild-type sample. Tumor tissues from surgical specimens of primary colon carcinoma (B) and its corresponding lymphatic metastasis (D) and hepatic metastasis (F). Tumor tissues from the third generation of xenograft models of primary colon carcinoma (C) and its corresponding lymphatic metastasis (E) and hepatic metastasis (G). (TIF) [file pone.0028384.s003.tif]

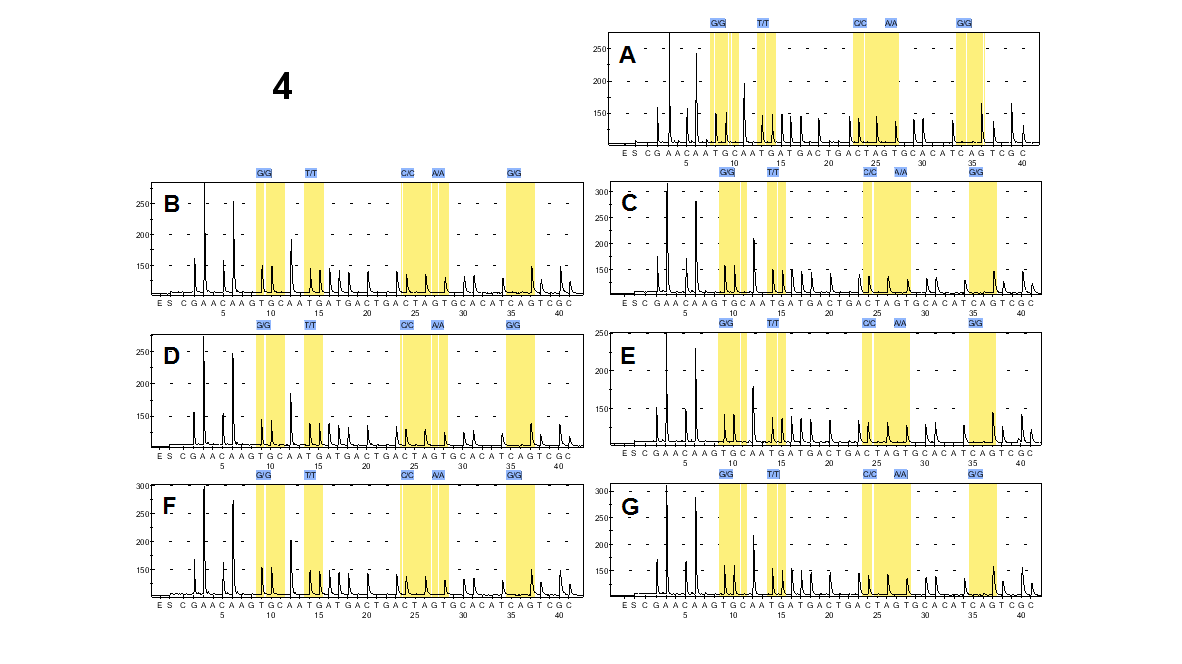

Supplement: Figure S4 — Mutation analysis by pyrosequencing. Sequence data of PIK3CA gene in exon 20 at codons 1043, 1044, 1047 and 1049 of primary tumor tissues and their early-generation xenograft models tumor tissues. A, standard wild-type sample. Tumor tissues from surgical specimens of primary colon carcinoma (B) and its corresponding lymphatic metastasis (D) and hepatic metastasis (F). Tumor tissues from the third generation of xenograft models of primary colon carcinoma (C) and its corresponding lymphatic metastasis (E) and hepatic metastasis (G). (TIF) [file pone.0028384.s004.tif]
