# Supplementary material for: Assessment of a Novel VEGF Targeted Agent Using Patient-Derived Tumor Tissue Xenograft Models of Colon Carcinoma with Lymphatic and Hepatic Metastases
Source: PLoS One. 2011 Dec 2;6(12):e28384. doi: 10.1371/journal.pone.0028384 (PMC3229582; doi:10.1371/journal.pone.0028384)
Supplement: Table S1 — Genes differentially expressed in patient primary colon cancer specimens and its xenograft. (DOC) [file pone.0028384.s005.doc]

**Table S1** Genes differentially expressed in patient primary colon cancer specimens and its xenograft

| **Probe Set ID** | **Regulation** | **Gene Symbol** | **Entrez Gene** | **Gene Title** |
| --- | --- | --- | --- | --- |
| 201744_s_at | down | LUM | 4060 | lumican |
| 204010_s_at | up | KRAS | 3845 | v-Ki-ras2 Kirsten rat sarcoma viral oncogene homolog |
| 204834_at | down | FGL2 | 10875 | fibrinogen-like 2 |
| 204845_s_at | up | ENPEP | 2028 | glutamyl aminopeptidase (aminopeptidase A) |
| 205886_at | down | REG1B | 5968 | regenerating islet-derived 1 beta |
| 205975_s_at | down | HOXD1 | 3231 | homeobox D1 |
| 206091_at | down | MATN3 | 4148 | matrilin 3 |
| 206579_at | up | ZNF192 | 7745 | zinc finger protein 192 |
| 207331_at | down |  |  |  |
| 207565_s_at | up | MR1 | 3140 | major histocompatibility complex, class I-related |
| 208348_s_at | up | CBLB | 868 | Cas-Br-M (murine) ecotropic retroviral transforming sequence b |
| 209752_at | down | REG1A | 5967 | regenerating islet-derived 1 alpha |
| 210306_at | down | L3MBTL | 26013 | l(3)mbt-like (Drosophila) |
| 211302_s_at | up | PDE4B | 5142 | phosphodiesterase 4B, cAMP-specific (phosphodiesterase E4 dunce homolog, Drosophila) |
| 211343_s_at | up | COL13A1 | 1305 | collagen, type XIII, alpha 1 |
| 212768_s_at | down | OLFM4 | 10562 | olfactomedin 4 |
| 213261_at | down | LBA1 | 9881 | lupus brain antigen 1 |
| 213908_at | up | WHAMML1 /// WHAMML2 | 339005 /// 440253 | WAS protein homolog associated with actin, golgi membranes and microtubules-like 1  /// WAS protein homolog associated with actin, golgi membranes and microtubules-like 2 (pseudogene) |
| 214341_at | down | THTPA | 79178 | Thiamine triphosphatase |
| 214409_at | up | RFPL3S | 10737 | RFPL3 antisense RNA (non-protein coding) |
| 214478_at | up | SPP2 | 6694 | secreted phosphoprotein 2, 24kDa |
| 214767_s_at | up | HSPB6 | 126393 | heat shock protein, alpha-crystallin-related, B6 |
| 214952_at | down | NCAM1 | 4684 | neural cell adhesion molecule 1 |
| 215902_at | up |  |  |  |
| 216613_at | down |  |  |  |
| 217966_s_at | down | FAM129A | 116496 | family with sequence similarity 129, member A |
| 218790_s_at | down | TMLHE | 55217 | trimethyllysine hydroxylase, epsilon |
| 219728_at | down | MYOT | 9499 | myotilin |
| 219947_at | up | CLEC4A | 50856 | C-type lectin domain family 4, member A |
| 220253_s_at | up | LRP12 | 29967 | low density lipoprotein-related protein 12 |
| 220669_at | down | OTUD4 | 54726 | OTU domain containing 4 |
| 220729_at | up |  |  |  |
| 221461_at | down | TAS2R9 | 50835 | taste receptor, type 2, member 9 |
| 222166_at | up | C9orf16 | 79095 | chromosome 9 open reading frame 16 |
| 222717_at | down | SDPR | 8436 | serum deprivation response (phosphatidylserine binding protein) |
| 222720_x_at | up | C1orf27 | 54953 | chromosome 1 open reading frame 27 |
| 223796_at | up | CNTNAP3 /// LOC643827 | 643827 /// 79937 | contactin associated protein-like 3 /// similar to cell recognition molecule CASPR3 |
| 223814_at | down | TRNT1 | 51095 | tRNA nucleotidyl transferase, CCA-adding, 1 |
| 223997_at | up | FNIP1 | 96459 | folliculin interacting protein 1 |
| 224253_at | up | EXOC5 | 10640 | exocyst complex component 5 |
| 224533_s_at | down | C6orf142 | 90523 | Chromosome 6 open reading frame 142 |
| 226057_at | up | CDGAP | 57514 | Cdc42 GTPase-activating protein |
| 226435_at | up | PAPLN | 89932 | papilin, proteoglycan-like sulfated glycoprotein |
| 227486_at | up | NT5E | 4907 | 5'-nucleotidase, ecto (CD73) |
| 228255_at | up | ALS2CR4 | 65062 | amyotrophic lateral sclerosis 2 (juvenile) chromosome region, candidate 4 |
| 228335_at | up | CLDN11 | 5010 | claudin 11 |
| 228459_at | up | FAM84A | 151354 | family with sequence similarity 84, member A |
| 228715_at | down | ZCCHC12 | 170261 | zinc finger, CCHC domain containing 12 |
| 229480_at | up |  |  |  |
| 229495_at | up | PM20D2 | 135293 | Peptidase M20 domain containing 2 |
| 229809_at | up | POU6F1 | 5463 | POU class 6 homeobox 1 |
| 229953_x_at | up | LCA5 | 167691 | Leber congenital amaurosis 5 |
| 230035_at | up | BOC | 91653 | Boc homolog (mouse) |
| 230045_at | down | CNTN2 | 6900 | contactin 2 (axonal) |
| 230512_x_at | up | TMEM165 | 55858 | Transmembrane protein 165 |
| 230736_at | down | LOC387647 | 387647 | patched domain containing 3 pseudogene |
| 231164_at | down | ABCA17P | 650655 | ATP-binding cassette, sub-family A (ABC1), member 17 (pseudogene) |
| 231356_at | up | LOC100131014 | 100131014 | similar to hCG2045213 |
| 231738_at | up | PCDHB7 | 56129 | protocadherin beta 7 |
| 232090_at | down | LOC100128178 | 100128178 | similar to hCG2041313 |
| 233109_at | up | COL12A1 | 1303 | Collagen, type XII, alpha 1 |
| 233578_at | up | C1orf101 | 257044 | chromosome 1 open reading frame 101 |
| 233797_s_at | down | CST11 | 140880 | cystatin 11 |
| 233871_at | down |  |  |  |
| 234259_at | down |  |  |  |
| 234442_at | up |  |  |  |
| 234667_at | down |  |  |  |
| 235079_at | down |  |  |  |
| 235696_at | up |  |  |  |
| 235976_at | up | SLITRK6 | 84189 | SLIT and NTRK-like family, member 6 |
| 236365_at | up |  |  |  |
| 236538_at | up | GRIA2 | 2891 | glutamate receptor, ionotropic, AMPA 2 |
| 236559_at | down | YWHAH | 7533 | Tyrosine 3-monooxygenase/tryptophan 5-monooxygenase activation protein, eta polypeptide |
| 236993_at | down |  |  |  |
| 237363_at | down | C9orf68 | 55064 | chromosome 9 open reading frame 68 |
| 237479_at | up |  |  |  |
| 237633_at | down |  |  |  |
| 237751_x_at | down |  |  |  |
| 237905_at | up | KRT25 | 147183 | keratin 25 |
| 238301_at | down |  |  |  |
| 238521_at | up |  |  |  |
| 240035_at | up |  |  |  |
| 240512_x_at | up | KCTD4 | 386618 | potassium channel tetramerisation domain containing 4 |
| 240712_s_at | down |  |  |  |
| 240852_at | up |  |  |  |
| 241098_at | up | CLEC7A | 64581 | C-type lectin domain family 7, member A |
| 241330_x_at | down |  |  |  |
| 241553_at | up | GSG1 | 83445 | germ cell associated 1 |
| 241643_at | up | TLK1 | 9874 | Tousled-like kinase 1 |
| 241749_at | up | MURC | 347273 | muscle-related coiled-coil protein |
| 242301_at | up | CBLN2 | 147381 | cerebellin 2 precursor |
| 242420_at | up |  |  |  |
| 242840_at | down |  |  |  |
| 243122_at | up |  |  |  |
| 243163_at | up |  |  |  |
| 243663_at | up |  |  |  |
| 243746_at | up | IGHMBP2 | 3508 | immunoglobulin mu binding protein 2 |
| 243799_x_at | up | ANGPTL3 | 27329 | Angiopoietin-like 3 |
| 244441_at | down |  |  |  |
| 244509_at | up | GPR155 | 151556 | G protein-coupled receptor 155 |
| 244643_at | up |  |  |  |
| 244835_at | down | C16orf52 | 730094 | Chromosome 16 open reading frame 52 |
| 1552315_at | up | GIMAP1 | 170575 | GTPase, IMAP family member 1 |
| 1552952_at | down | RBMY2FP | 159162 | RNA binding motif protein, Y-linked, family 2, member F pseudogene |
| 1554176_a_at | up | C3orf33 | 285315 | chromosome 3 open reading frame 33 |
| 1553914_at | up | MGC34800 | 162137 | hypothetical protein MGC34800 |
| 1555053_at | up | SYT9 | 143425 | synaptotagmin IX |
| 1555394_at | up | PIGK | 10026 | phosphatidylinositol glycan anchor biosynthesis, class K |
| 1555717_at | down |  |  |  |
| 1555774_at | down | ZAR1 | 326340 | zygote arrest 1 |
| 1556008_a_at | up |  |  |  |
| 1556798_a_at | up | LOC386597 | 386597 | hypothetical protein LOC386597 |
| 1556828_at | up |  |  |  |
| 1557107_at | up | LOC286002 | 286002 | hypothetical protein LOC286002 |
| 1557881_at | up | C10orf44 | 414201 | chromosome 10 open reading frame 44 |
| 1557921_s_at | up |  |  |  |
| 1558234_at | up | FLJ36644 | 400617 | hypothetical protein LOC400617 |
| 1558480_at | up |  |  |  |
| 1558525_at | up |  |  |  |
| 1559788_at | up |  |  |  |
| 1561114_a_at | up | DEPDC4 | 120863 | DEP domain containing 4 |
| 1560144_at | down |  |  |  |
| 1560458_s_at | up | CAPS2 | 84698 | calcyphosine 2 |
| 1560898_at | down |  |  |  |
| 1560944_at | up | FLJ40434 | 163742 | hypothetical FLJ40434 |
| 1561260_at | down |  |  |  |
| 1561288_at | up |  |  |  |
| 1561432_at | down |  |  |  |
| 1561906_at | up |  |  |  |
| 1562472_at | up |  |  |  |
| 1564276_at | down | C5orf56 | 441108 | chromosome 5 open reading frame 56 |
| 1564469_at | up | LMOD3 | 56203 | leiomodin 3 (fetal) |
| 1566158_at | up |  |  |  |
| 1566266_at | down |  |  |  |
| 1566295_at | up |  |  |  |
| 1566551_at | down |  |  |  |
| 1569240_at | up | ZNF93 | 81931 | zinc finger protein 93 |
| 1569953_at | up |  |  |  |
| 1570298_at | down |  |  |  |
| 1570528_at | up | XYLT2 | 64132 | xylosyltransferase II |
