# Supplementary material for: Assessment of a Novel VEGF Targeted Agent Using Patient-Derived Tumor Tissue Xenograft Models of Colon Carcinoma with Lymphatic and Hepatic Metastases
Source: PLoS One. 2011 Dec 2;6(12):e28384. doi: 10.1371/journal.pone.0028384 (PMC3229582; doi:10.1371/journal.pone.0028384)
Supplement: Table S2 — Genes differentially expressed in patient colon cancer lymphatic metastasis specimens and its xenograft. (DOC) [file pone.0028384.s006.doc]

**Table S2** Genes differentially expressed in patient colon cancer lymphatic metastasis specimens and its xenograft

| **Probe Set ID** | **Regulation** | **Gene Symbol** | **Entrez Gene** | **Gene Title** |
| --- | --- | --- | --- | --- |
| 203680_at | down | PRKAR2B | 5577 | protein kinase, cAMP-dependent, regulatory, type II, beta |
| 205741_s_at | up | DTNA | 1837 | dystrobrevin, alpha |
| 205994_at | down | ELK4 | 2005 | ELK4, ETS-domain protein (SRF accessory protein 1) |
| 206101_at | down | ECM2 | 1842 | extracellular matrix protein 2, female organ and adipocyte specific |
| 208394_x_at | up | ESM1 | 11082 | endothelial cell-specific molecule 1 |
| 208481_at | up | ASB4 | 51666 | ankyrin repeat and SOCS box-containing 4 |
| 209125_at | up | KRT6A | 3853 | keratin 6A |
| 211019_s_at | down | LSS | 4047 | lanosterol synthase (2,3-oxidosqualene-lanosterol cyclase) |
| 211959_at | down | IGFBP5 | 3488 | insulin-like growth factor binding protein 5 |
| 212298_at | down | NRP1 | 8829 | neuropilin 1 |
| 214319_at | down | FRY | 10129 | furry homolog (Drosophila) |
| 215306_at | down |  |  |  |
| 215972_at | down |  |  |  |
| 216498_at | up |  |  |  |
| 217546_at | up | MT1M | 4499 | metallothionein 1M |
| 219768_at | up | VTCN1 | 79679 | V-set domain containing T cell activation inhibitor 1 |
| 220401_at | down | FLJ21369 | 79860 | hypothetical protein FLJ21369 |
| 222668_at | up | KCTD15 | 79047 | potassium channel tetramerisation domain containing 15 |
| 223932_at | up |  |  |  |
| 223933_at | down | KIF5A | 3798 | kinesin family member 5A |
| 227361_at | down | HS3ST3B1 | 9953 | heparan sulfate (glucosamine) 3-O-sulfotransferase 3B1 |
| 228057_at | down | DDIT4L | 115265 | DNA-damage-inducible transcript 4-like |
| 228335_at | up | CLDN11 | 5010 | claudin 11 |
| 229228_at | up | CREB5 | 9586 | cAMP responsive element binding protein 5 |
| 229518_at | up | FAM46B | 115572 | family with sequence similarity 46, member B |
| 230319_at | down |  |  |  |
| 230487_at | up | C6orf99 /// LOC100130967 | 100130967 /// 389440 | chromosome 6 open reading frame 99 /// similar to hCG2044932 |
| 230784_at | down | PRAC | 84366 | prostate cancer susceptibility candidate |
| 233372_at | up |  |  |  |
| 233902_at | up | GUCA1C | 9626 | guanylate cyclase activator 1C |
| 234671_at | down | KRTAP4-2 | 85291 | keratin associated protein 4-2 |
| 236138_at | down |  |  |  |
| 236468_at | down |  |  |  |
| 236910_at | down | MRPL39 | 54148 | Mitochondrial ribosomal protein L39 |
| 237996_at | up |  |  |  |
| 239698_at | up |  |  |  |
| 239871_at | down | CLTC | 1213 | Clathrin, heavy chain (Hc) |
| 240049_at | up |  |  |  |
| 240539_at | up |  |  |  |
| 240620_at | down |  |  |  |
| 241044_x_at | up |  |  |  |
| 241868_at | down |  |  |  |
| 241891_at | down |  |  |  |
| 241926_s_at | down | ERG | 2078 | v-ets erythroblastosis virus E26 oncogene homolog (avian) |
| 242316_at | up |  |  |  |
| 242638_at | up |  |  |  |
| 242870_at | up | RIMKLB | 57494 | Ribosomal modification protein rimK-like family member B |
| 243163_at | up |  |  |  |
| 243566_at | up |  |  |  |
| 1552580_at | up | TRIML2 | 205860 | tripartite motif family-like 2 |
| 1552691_at | down | ARL11 | 115761 | ADP-ribosylation factor-like 11 |
| 1552947_x_at | down | ZNF114 | 163071 | zinc finger protein 114 |
| 1554864_a_at | up | SDC3 | 9672 | syndecan 3 |
| 1554918_a_at | down | ABCC4 | 10257 | ATP-binding cassette, sub-family C (CFTR/MRP), member 4 |
| 1556200_a_at | up | C10orf90 | 118611 | chromosome 10 open reading frame 90 |
| 1556777_a_at | up |  |  |  |
| 1556827_at | down | LOC339929 | 339929 | hypothetical protein LOC339929 |
| 1556912_at | up | GIT2 | 9815 | G protein-coupled receptor kinase interacting ArfGAP 2 |
| 1559045_at | down | LOC100128288 | 100128288 | hypothetical protein LOC100128288 |
| 1560888_x_at | down |  |  |  |
| 1561573_at | up |  |  |  |
| 1561817_at | up |  |  |  |
| 1562053_at | down |  |  |  |
| 1562130_at | down | IQCA1 | 79781 | IQ motif containing with AAA domain 1 |
| 1562470_at | up | C1orf83 | 127428 | chromosome 1 open reading frame 83 |
| 1562572_at | down |  |  |  |
| 1563612_at | up |  |  |  |
| 1569408_at | up | EIF2C4 | 192670 | Eukaryotic translation initiation factor 2C, 4 |
| 1569450_at | down | CAPZA2 | 830 | capping protein (actin filament) muscle Z-line, alpha 2 |
| 1570444_at | down | LOC643201 | 643201 | hypothetical protein LOC643201 |
