# Supplementary material for: Assessment of a Novel VEGF Targeted Agent Using Patient-Derived Tumor Tissue Xenograft Models of Colon Carcinoma with Lymphatic and Hepatic Metastases
Source: PLoS One. 2011 Dec 2;6(12):e28384. doi: 10.1371/journal.pone.0028384 (PMC3229582; doi:10.1371/journal.pone.0028384)
Supplement: Table S3 — Genes differentially expressed in patient colon cancer hepatic metastasis specimens and its xenograft. (DOC) [file pone.0028384.s007.doc]

**Table S3** Genes differentially expressed in patient colon cancer hepatic metastasis specimens and its xenograft

| **Probe Set ID** | **Regulation** | **Gene Symbol** | **Entrez Gene** | **Gene Title** |
| --- | --- | --- | --- | --- |
| 203561_at | down | FCGR2A | 2212 | Fc fragment of IgG, low affinity IIa, receptor (CD32) |
| 203676_at | down | GNS | 2799 | glucosamine (N-acetyl)-6-sulfatase |
| 205815_at | up | REG3A | 5068 | regenerating islet-derived 3 alpha |
| 205886_at | up | REG1B | 5968 | regenerating islet-derived 1 beta |
| 206218_at | up | MAGEB2 | 4113 | melanoma antigen family B, 2 |
| 207995_s_at | down | CLEC4M | 10332 | C-type lectin domain family 4, member M |
| 208250_s_at | up | DMBT1 | 1755 | deleted in malignant brain tumors 1 |
| 209752_at | up | REG1A | 5967 | regenerating islet-derived 1 alpha |
| 210631_at | down | NF1 | 4763 | neurofibromin 1 |
| 211241_at | down | ANXA2P3 | 305 | annexin A2 pseudogene 3 |
| 213472_at | down | HNRNPH1 | 3187 | heterogeneous nuclear ribonucleoprotein H1 (H) |
| 213660_s_at | up | TOP3B | 8940 | topoisomerase (DNA) III beta |
| 214059_at | up | IFI44 | 10561 | Interferon-induced protein 44 |
| 214397_at | down | MBD2 | 8932 | methyl-CpG binding domain protein 2 |
| 214417_s_at | down | FETUB | 26998 | Fetuin B |
| 214834_at | down | PAR5 | 8123 | Prader-Willi/Angelman syndrome-5 |
| 214914_at | up | FAM13C | 220965 | family with sequence similarity 13, member C |
| 215172_at | down | PTPN20A /// PTPN20B | 26095 /// 653129 | protein tyrosine phosphatase, non-receptor type 20A /// protein tyrosine phosphatase, non-receptor type 20B |
| 215512_at | down | 6-Mar | 10299 | membrane-associated ring finger (C3HC4) 6 |
| 216009_at | up | SLC39A9 | 55334 | Solute carrier family 39 (zinc transporter), member 9 |
| 216926_s_at | down | KIAA0892 | 23383 | KIAA0892 |
| 217080_s_at | up | HOMER2 | 9455 | homer homolog 2 (Drosophila) |
| 217101_at | down | TTC39A | 22996 | tetratricopeptide repeat domain 39A |
| 217105_at | up |  |  |  |
| 217292_at | up | MTMR7 | 9108 | myotubularin related protein 7 |
| 217321_x_at | down | ATXN3 | 4287 | Ataxin 3 |
| 217549_at | down |  |  |  |
| 217552_x_at | down | CR1 | 1378 | complement component (3b/4b) receptor 1 (Knops blood group) |
| 219761_at | down | CLEC1A | 51267 | C-type lectin domain family 1, member A |
| 219937_at | down | TRHDE | 29953 | thyrotropin-releasing hormone degrading enzyme |
| 220550_at | down | FBXO4 | 26272 | F-box protein 4 |
| 222281_s_at | down |  |  |  |
| 222305_at | up | HK2 | 3099 | hexokinase 2 |
| 222314_x_at | down | EGO | 100126791 | eosinophil granule ontogeny |
| 222453_at | up | CYBRD1 | 79901 | cytochrome b reductase 1 |
| 224029_x_at | up | SCN11A | 11280 | sodium channel, voltage-gated, type XI, alpha subunit |
| 224154_at | down | B3GNT2 | 10678 | UDP-GlcNAc:betaGal beta-1,3-N-acetylglucosaminyltransferase 2 |
| 224772_at | down | NAV1 | 89796 | neuron navigator 1 |
| 226344_at | down | ZMAT1 | 84460 | zinc finger, matrin type 1 |
| 227623_at | down |  |  |  |
| 228534_s_at | down |  |  |  |
| 228800_x_at | up | AURKAIP1 | 54998 | aurora kinase A interacting protein 1 |
| 229352_at | down | SPESP1 | 246777 | sperm equatorial segment protein 1 |
| 229725_at | up | ACSL6 | 23305 | Acyl-CoA synthetase long-chain family member 6 |
| 229976_at | down | MORN5 | 254956 | MORN repeat containing 5 |
| 230964_at | down | FREM2 | 341640 | FRAS1 related extracellular matrix protein 2 |
| 231485_at | up |  |  |  |
| 231544_s_at | down | POLR3G | 10622 | Polymerase (RNA) III (DNA directed) polypeptide G (32kD) |
| 231673_at | down |  |  |  |
| 231969_at | down | STOX2 | 56977 | storkhead box 2 |
| 232049_at | down |  |  |  |
| 232691_at | down | SFXN5 | 94097 | sideroflexin 5 |
| 232812_at | down | LOC401052 | 401052 | hypothetical LOC401052 |
| 232881_at | up | GNASAS | 149775 | GNAS antisense RNA (non-protein coding) |
| 233086_at | down | C20orf106 /// C20orf107 | 200232 /// 388799 | chromosome 20 open reading frame 106 /// chromosome 20 open reading frame 107 |
| 233351_at | down |  |  |  |
| 233367_at | down |  |  |  |
| 233707_at | up |  |  |  |
| 233739_at | up |  |  |  |
| 234196_at | down |  |  |  |
| 234220_at | down | CADPS | 8618 | Ca++-dependent secretion activator |
| 234472_at | down | GALNT13 | 114805 | UDP-N-acetyl-alpha-D-galactosamine:polypeptide N-acetylgalactosaminyltransferase 13 (GalNAc-T13) |
| 234789_at | down |  |  |  |
| 234851_at | down | IGL@ | 3535 | Immunoglobulin lambda locus |
| 234861_at | down | LOC93463 | 93463 | hypothetical protein LOC93463 |
| 234990_at | down | CBX5 | 23468 | chromobox homolog 5 (HP1 alpha homolog, Drosophila) |
| 235334_at | down | ST6GALNAC3 | 256435 | ST6 (alpha-N-acetyl-neuraminyl-2,3-beta-galactosyl-1,3)-N-acetylgalactosaminide alpha-2,6-sialyltransferase 3 |
| 235696_at | up |  |  |  |
| 235951_s_at | down | ZNF688 | 146542 | zinc finger protein 688 |
| 236627_at | down |  |  |  |
| 237066_at | down |  |  |  |
| 237375_at | down | LOC100131480 | 100131480 | similar to hCG2045844 |
| 237516_at | down |  |  |  |
| 237608_at | down |  |  |  |
| 238305_at | down |  |  |  |
| 238362_at | up |  |  |  |
| 238399_x_at | up |  |  |  |
| 238634_x_at | up |  |  |  |
| 238757_at | down | DBF4B | 80174 | DBF4 homolog B (S. cerevisiae) |
| 238889_at | down | AGBL5 | 60509 | ATP/GTP binding protein-like 5 |
| 239563_at | down |  |  |  |
| 239872_at | down |  |  |  |
| 239883_s_at | down | ANO4 | 121601 | Anoctamin 4 |
| 239963_at | down |  |  |  |
| 239995_at | down |  |  |  |
| 240474_x_at | up |  |  |  |
| 240762_at | down |  |  |  |
| 240965_at | down |  |  |  |
| 240992_at | down |  |  |  |
| 241128_at | up |  |  |  |
| 241275_at | down | CAPZA1 | 829 | Capping protein (actin filament) muscle Z-line, alpha 1 |
| 241308_at | down | LOC729589 | 729589 | hypothetical LOC729589 |
| 241730_at | down |  |  |  |
| 241834_at | down | IPW | 3653 | imprinted in Prader-Willi syndrome (non-protein coding) |
| 242002_at | up | NKAIN2 | 154215 | Na+/K+ transporting ATPase interacting 2 |
| 242331_x_at | down | LOC642236 | 642236 | similar to FRG1 protein (FSHD region gene 1 protein) |
| 242340_at | down |  |  |  |
| 242559_at | down |  |  |  |
| 242628_at | down |  |  |  |
| 242821_at | down | C9orf93 | 203238 | chromosome 9 open reading frame 93 |
| 243320_at | down |  |  |  |
| 243641_at | up |  |  |  |
| 243660_at | down | CHD9 | 80205 | Chromodomain helicase DNA binding protein 9 |
| 243672_at | down | SALL3 | 27164 | Sal-like 3 (Drosophila) |
| 243689_s_at | down | LOC283788 | 283788 | FSHD region gene 1 pseudogene |
| 243925_at | down |  |  |  |
| 243998_at | down | KRT222P | 125113 | keratin 222 pseudogene |
| 244726_at | down |  |  |  |
| 1555687_a_at | down | CLEC4C | 170482 | C-type lectin domain family 4, member C |
| 1552900_a_at | down | RP4-662A9.2 | 154089 | hypothetical protein MGC34034 |
| 1553372_at | up | FLJ23865 | 200317 | hypothetical protein FLJ23865 |
| 1553711_a_at | down | C4orf39 | 152756 | chromosome 4 open reading frame 39 |
| 1554328_at | up | STXBP4 | 252983 | syntaxin binding protein 4 |
| 1555053_at | down | SYT9 | 143425 | synaptotagmin IX |
| 1555055_at | down | AVL9 | 23080 | AVL9 homolog (S. cerevisiase) |
| 1555217_at | up | UBE2W | 55284 | ubiquitin-conjugating enzyme E2W (putative) |
| 1556410_a_at | up | KRTAP19-1 | 337882 | keratin associated protein 19-1 |
| 1556474_a_at | down | FLJ38379 | 285097 | hypothetical FLJ38379 |
| 1556877_at | down |  |  |  |
| 1557736_at | down | NKTR | 4820 | natural killer-tumor recognition sequence |
| 1558636_s_at | down | ADAMTS5 | 11096 | ADAM metallopeptidase with thrombospondin type 1 motif, 5 |
| 1558682_at | down | HMGA2 | 8091 | high mobility group AT-hook 2 |
| 1559203_s_at | down | KRAS | 3845 | v-Ki-ras2 Kirsten rat sarcoma viral oncogene homolog |
| 1559353_at | down |  |  |  |
| 1559576_at | down | WDR78 | 79819 | WD repeat domain 78 |
| 1559770_at | down |  |  |  |
| 1559806_at | down |  |  |  |
| 1559807_at | down |  |  |  |
| 1559975_at | up | BTG1 | 694 | B-cell translocation gene 1, anti-proliferative |
| 1560033_at | down | MCCC2 | 64087 | methylcrotonoyl-Coenzyme A carboxylase 2 (beta) |
| 1560187_at | down |  |  |  |
| 1561445_at | down |  |  |  |
| 1561486_at | down |  |  |  |
| 1561521_at | down | S100B | 6285 | S100 calcium binding protein B |
| 1562236_at | down | MYST4 | 23522 | MYST histone acetyltransferase (monocytic leukemia) 4 |
| 1564444_at | down | LOC100130264 | 100130264 | hypothetical LOC100130264 |
| 1564841_at | down |  |  |  |
| 1565693_at | down | DTYMK | 1841 | Deoxythymidylate kinase (thymidylate kinase) |
| 1566632_at | down |  |  |  |
| 1566694_at | down |  |  |  |
| 1569108_a_at | down | ZNF589 | 51385 | zinc finger protein 589 |
| 1569669_at | up | FOXR2 | 139628 | forkhead box R2 |
| 1569730_at | down | HEATR7B2 | 133558 | HEAT repeat family member 7B2 |
| 1569881_at | up | DRP2 | 1821 | dystrophin related protein 2 |
| 1570098_at | down |  |  |  |
